# Elucidation of population-based bacterial adaptation to antimicrobial treatment by single-cell sequencing analysis of the gut microbiome of a hospital patient

**DOI:** 10.1128/msystems.01631-24

**Published:** 2025-12-30

**Authors:** Lianwei Ye, Yuchen Wu, Jiubiao Guo, Hanyu Wang, Jing Cai, Kaichao Chen, Ning Dong, Jiale Yu, Shan Chao, Hongwei Zhou, Gongxiang Chen, Sheng Chen, Rong Zhang

**Affiliations:** 1 Department of Clinical Laboratory, Second Affiliated Hospital of Zhejiang University, School of Medicine, Hangzhou, China; 2 Department of Infectious Diseases and Public Health, Jockey Club College of Veterinary Medicine and Life Sciences, City University of Hong Kong Kowloon 53025, Hong Kong, Hong Kong; 3 State Key Laboratory of Chemical Biology and Drug Discovery and the Department of Food Science and Nutrition, The Hong Kong Polytechnic University 26680, Hong Kong, Hong Kong; 4 Clinical Research Center, The First Affiliated Hospital of Shantou University Medical College 485866, Shantou, China; 5 Neuroscience Intensive Care Unit, Second Affiliated Hospital of Zhejiang University, School of Medicine, Hangzhou, China; 6 School of Public Health, Zhejiang University School of Medicine 26441, Hangzhou, People's Republic of China; 7 MobiDrop (Zhejiang) Co., Ltd, Shanghai, China; 8 Shenzhen Key Laboratory for Food Biological Safety Control, The Hong Kong Polytechnic University Shenzhen Research Institute 597660, , Shenzhen, China; California State University Stanislaus, Turlock, California, USA

**Keywords:** single-cell sequencing, gut microbiome, antibiotic resistance genes (ARGs), horizontal gene transfer (HGT), *Klebsiella pneumoniae*

## Abstract

In this study, we used single-cell sequencing to analyze the gut microbiome of an adult male patient with acute cerebral hemorrhage undergoing antibiotic treatment. We identified 92 bacterial species, including 23 Firmicutes and one archaeon from Methanobacteriota, along with 69 unclassified strains. Single-cell sequencing effectively detected bacteria carrying antibiotic resistance genes (ARGs), particularly in unclassified species, and traced the evolution of these genes across diverse bacterial taxa. Notably, the *cfr(C*) gene was detected in 11 bacterial species following antimicrobial treatment, with mutation patterns characterized in *Enterococcus faecalis*, *Klebsiella pneumoniae*, *Ruthenibacterium UN-1*, and four unclassified species. In total, 29 ARG subtypes across eight types were identified in 13 known, five unknown, and 18 unclassified species, allowing us to trace their evolution routes. In addition, we detected a total of 309 horizontal gene transfer (HGT) events, in which several genes like *folE* and *queE* were frequently involved. The products of these genes are known to enhance the ability of the recipient bacterial strains to repair DNA damage and maintain genomic stability, especially following prolonged antibiotic treatment. Comparison between isolated strain genomes (IS-KP1) and single-cell analysis confirmed the presence of at least two *K. pneumoniae* strains in the patient, with one exhibiting a larger extent of involvement in ARG co-evolution. This strain was found to contain the *cfr(C*) and *fosXCC* genes, which were absent in IS-KP1. *Klebsiella* strains were also found to participate actively in HGT events. In conclusion, the study identified a wide range of ARGs and HGT events within the microbiome. The detection of *K. pneumoniae* strains with distinct ARG evolution patterns underscores the gut microbiome’s adaptability to environmental changes. These findings facilitate the development of novel antimicrobial strategies by fine-tuning the gut microbiome composition.

**IMPORTANCE:**

This study highlights the power of single-cell sequencing to unravel the diversity and dynamics of the gut microbiome during antibiotic treatment in a patient with acute cerebral hemorrhage. By identifying antibiotic resistance genes (ARGs) in both known and unclassified bacterial species, we reveal the intricate evolution and horizontal transfer of resistance traits across taxa. The discovery of distinct ARG patterns, including the emergence of the *cfr(C*) gene in multiple species and its co-evolution in *K. pneumoniae*, underscores the gut microbiome’s adaptability to antimicrobial pressures. These findings provide critical insights into the mechanisms driving resistance dissemination and offer potential pathways for developing precision microbiome-based therapies to combat antibiotic resistance.

## INTRODUCTION

The human intestine harbors a vast array of microorganisms, including bacteria, viruses, fungi, and archaea. These microorganisms play a significant role in human health, aiding digestion and warding off pathogenic invaders (1–3). On the flip side, the gut microbiota may harbor numerous genes, such as antibiotic resistance genes (ARGs) and virulence factor genes (VFGs), that enable potential bacterial pathogens to exhibit antibiotic resistance and the ability to cause infection. Experimental evidence gathered in recent studies suggests that significant changes occur in the gut microbiota following drug treatments, the extent of which requires further studies to ascertain their implications (4, 5). In simpler terms, the gut microbiota can carry genes that help bacteria resist antibiotics (ARGs) or cause infections (virulence genes). When people take antibiotic treatment, these treatments can disturb the balance of the gut microbes and increase the risk of drug-resistant or harmful bacteria growing. More studies are needed to understand how these changes affect human health. Hence, studying the gut microbiota holds great significance in devising new strategies for disease control through boosting immunity and mitigating dissemination of potential pathogens in the human body. On the other hand, there is increasing concern over settlement of pathogenic bacteria carrying drug-resistance genes within the gut microbiota. Not only do these pathogenic strains pose a threat to human health by causing opportunistic infection, but they also render commonly used antibiotics ineffective through carriage and dissemination of ARGs. Among them, CR-KP, or carbapenem-resistant *K. pneumoniae*, is a major pathogen known to cause a wide range of infections, including pneumonia, urinary tract infections, and bloodstream infections (6–8).

Traditional research techniques such as bacterial culture and metagenomic sequencing have their own advantages and limitations in the analysis of the structure of the human gut microbiome. Bacterial culture allows for the isolation of specific strains and their genomes. The organisms can then be tested in *in vitro* and animal experiments to validate the functional characteristics of specific strains such as drug resistance and pathogenicity (9–12). This approach can generate valuable data that facilitate development of preventive and therapeutic strategies. However, the main drawback of this method lies in its limitation to study only a small number of cultivable microorganisms. On the other hand, metagenomic sequencing offers the advantage of studying the entire microbial community, not only identifying the range of bacteria present but also depicting the range of functional genes that exist within the entire microbiome, including ARGs, VFGs, and those that encode various metabolic functions. Moreover, through metagenome-assembled genomes (MAGs) (13–16), unculturable and unclassified genomes within the human gut microbiome can be identified. Nonetheless, a plethora of genetically uncharacterized microorganisms exist in the human microbiome. It is necessary to determine the types of ARGs, VFGs, and other functional genes harbored by different bacterial species and characterize the horizontal gene transfer (HGT) events that occur among members of the gut microbiome.

Recent advancements in single-cell sequencing technologies have revolutionized our ability to explore the genetic landscape of microbial communities at unprecedented resolution (17–19). By isolating and sequencing individual bacterial cells, researchers can now delve into the genetic makeup of previously unknown species that inhabit the gut environment. In addition, this approach enhances our understanding of HGT activities among strains of different bacterial species, including exchanges between unclassified and uncultivable bacteria or archaea.

In this study, we conducted an in-depth investigation of the composition and characteristics of a patient’s gut microbiome following antibiotic treatment. Our main scientific question was: How does antibiotic treatment influence the co-evolution and transfer of ARGs and virulence genes in the human gut microbiome at the single-cell level? To address this, we leveraged a combination of single-cell sequencing, metagenomics, and comparative genomics to reveal strain-level variations in ARGs, VFGs, and plasmid profiles. We also delineated the network of HGT events that occurred among known and previously unidentified bacterial taxa. Our findings highlight the power of single-cell analysis in uncovering novel bacterial species and the genomic mechanisms driving the emergence and spread of antibiotic resistance in clinically relevant microbiota.

## RESULTS

### Gut microbiome composition at the single-cell level

**Fig 1:**
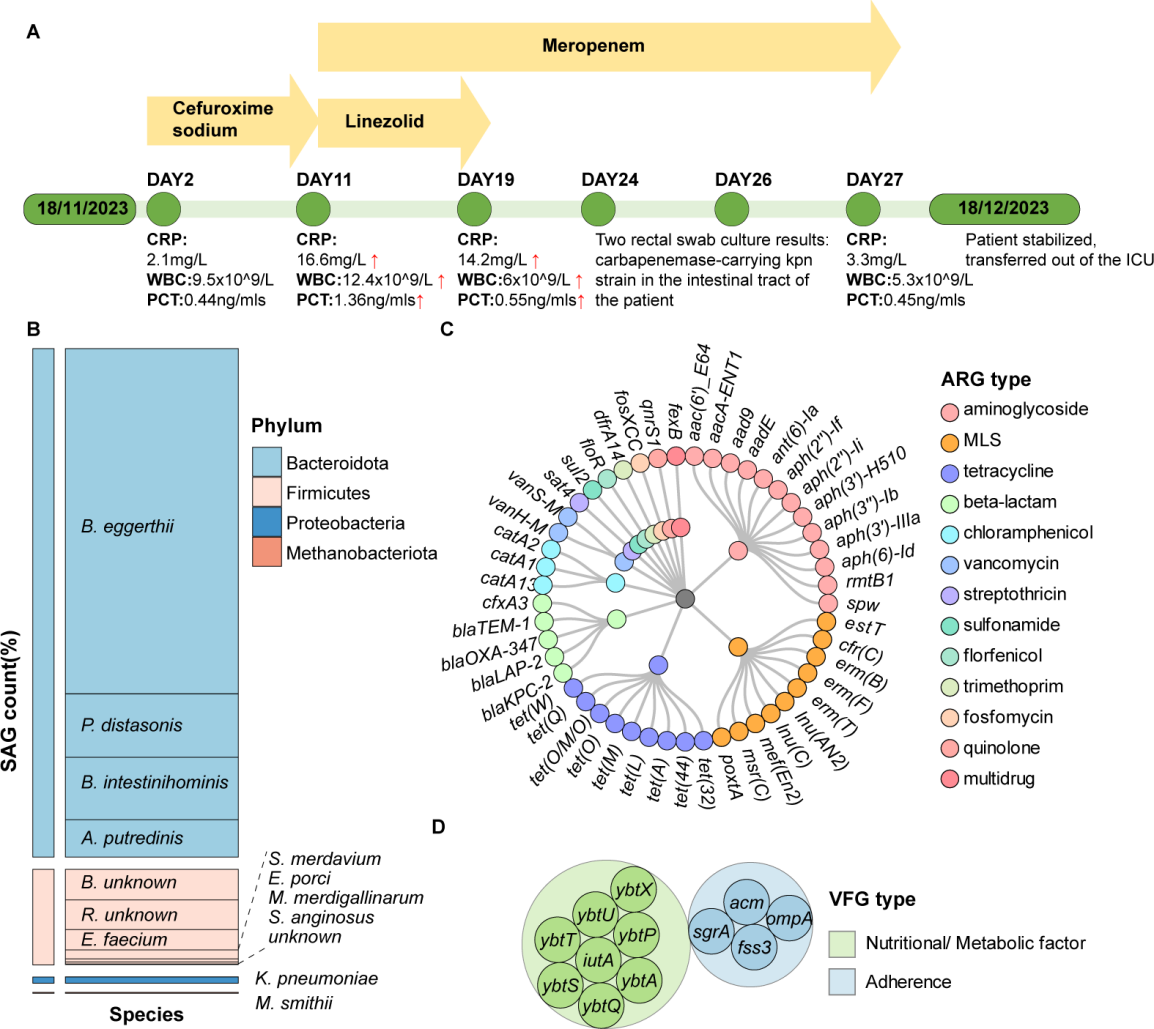
Analysis of variation in the composition of the gut microbiome of a patient subjected to serial antimicrobial treatment. (**A**) Timeline of treatment of the patient by various antibiotics. Key events, such as detection of carbapenem-resistant *K. pneumoniae* and stabilization of the condition of the patient, are highlighted. (**B**) Bar graph showing the abundance of different bacterial species in the patient’s microbiome, as determined by recording single amplified genome (SAG) counts. Each color represents a different bacterial species. (**C**) Circular diagram illustrating the diversity of antibiotic resistance gene (ARG) types and subtypes present in the microbiome. Each ARG type and subtype is color-coded for easy identification. (**D**) Representation of virulence factor genes (VFGs) identified in the microbiome, with a focus on genes associated with nutritional uptake, metabolic factors, and adherence functions.

We conducted an analysis of the composition of the gut microbiome of an adult male patient presented with acute cerebral hemorrhage; initial antimicrobial treatment encompassed intravenous administration of cefuroxime sodium given to this patient. Subsequently, meropenem and linezolid were administered upon the manifestation of fever and productive cough with sputum. Concurrently, a fecal sample was obtained for single-cell sequencing on 28 November 2023 during the antimicrobial treatment period (Fig. 1). Analysis of the patient’s gut microbiome showed that it was predominantly composed of three bacterial phyla—Bacteroidetes, Firmicutes, and Proteobacteria—along with Archaea from the phylum Methanobacteriota. Among them, Bacteroidetes and Firmicutes were predominant, constituting 83.2% and 15.6% of the single-amplified genomes (SAGs), respectively (Fig. 1; Tables S1 and S2). At the species level, certain taxa exhibited notably high prevalence, with *Bacteroides eggerthii* (Bacteroidetes) emerging as the predominant strain; this species accounted for as much as 56.5% of the microbial population. *Parabacteroides distasonis* and *B. intestinihominis* (both Bacteroidetes) were also abundant, comprising 10.4% and 10.2% of the microbial community, respectively. We were able to identify less abundant yet potentially clinically relevant species in the data set, including *Enterococcus faecium* (Firmicutes, 3.3%), *K. pneumoniae* (Proteobacteria, 1.0%), *Methanobrevibacter smithii* (Methanobacteriota, 0.2%), and *Streptococcus anginosus* (Firmicutes, 0.1%). Comparison with the metagenomic data revealed that *Methanobrevibacter smithii* (Methanobacteriota) was exclusively detected in the single-cell data set. Other bacterial species uniquely identified through single-cell analysis included *E. faecium*, *Scatomonas merdavium* (*Bacteroidetes*), *Eisenbergiella porci* (*Firmicutes*), *Massiliomicrobiota merdigallinarum* (*Firmicutes*), and *M. smithii*. These findings underscore the value of single-cell analysis in uncovering previously undetectable taxonomic groups within the gut microbiome.

A comprehensive investigation of ARGs within the gut microbiome revealed the presence of 13 distinct types, spanning various antibiotic classes (Fig. 1; Tables S3 and S4). Prominent among these ARGs were aminoglycoside resistance genes, which comprised 10 subtypes, along with prevalent beta-lactam resistance genes, including *bla*_KPC-2_ and *bla*_TEM-1_, and chloramphenicol resistance genes such as *cat*A13 and *cat*A14. Additional ARG classes, such as those that encode resistance to florfenicol, fosfomycin, macrolide–lincosamide–streptogramin (MLS), quinolone, streptothricin, sulfonamide, tetracycline, trimethoprim, and vancomycin, were found to involve a diverse array of resistance determinants.

Further analysis uncovered a rich diversity of subtypes within each ARG type, which is indicative of the genetic complexity of gut bacteria and their adaptability to pressures associated with antimicrobial treatment. For instance, the aminoglycoside resistance genes were found to comprise 13 subtypes, including *aph*(2'')-If and *aph* (6)-Id, illustrating that gut bacteria utilize a diverse range of mechanisms to counteract the action of this class of antibiotics. Tetracycline resistance genes had nine subtypes, including *tet*(32) and *tet*(M). Among the beta-lactam resistance genes, a myriad of subtypes were also identified. Noteworthy beta-lactam subtypes included *bla*_KPC-2_, and *bla*_LAP-2_, each exhibiting distinct mechanisms of resistance but all being clinically important. Notable ARGs exclusively identified in the single-cell data set included *aacA*-ENT1, *aph*(2'')-If, *aph*(2'')-Ii, *aph*(3')-H510, *floR*, *erm*(B), *erm*(F), *erm*(G), *est*T, *msr*(C), *tet*(32), *tet*(40), *tet*(44), *tet*(L), *tet*(M), *tet*(O/M/O), *tet*(Q), *van*H-M, and *van*S-M (Table S5), indicating that single-cell analysis can help produce high-resolution genetic features of members of the human gut microbiome.

### Bacterial hosts of ARGs and VFGs

**Fig 2:**
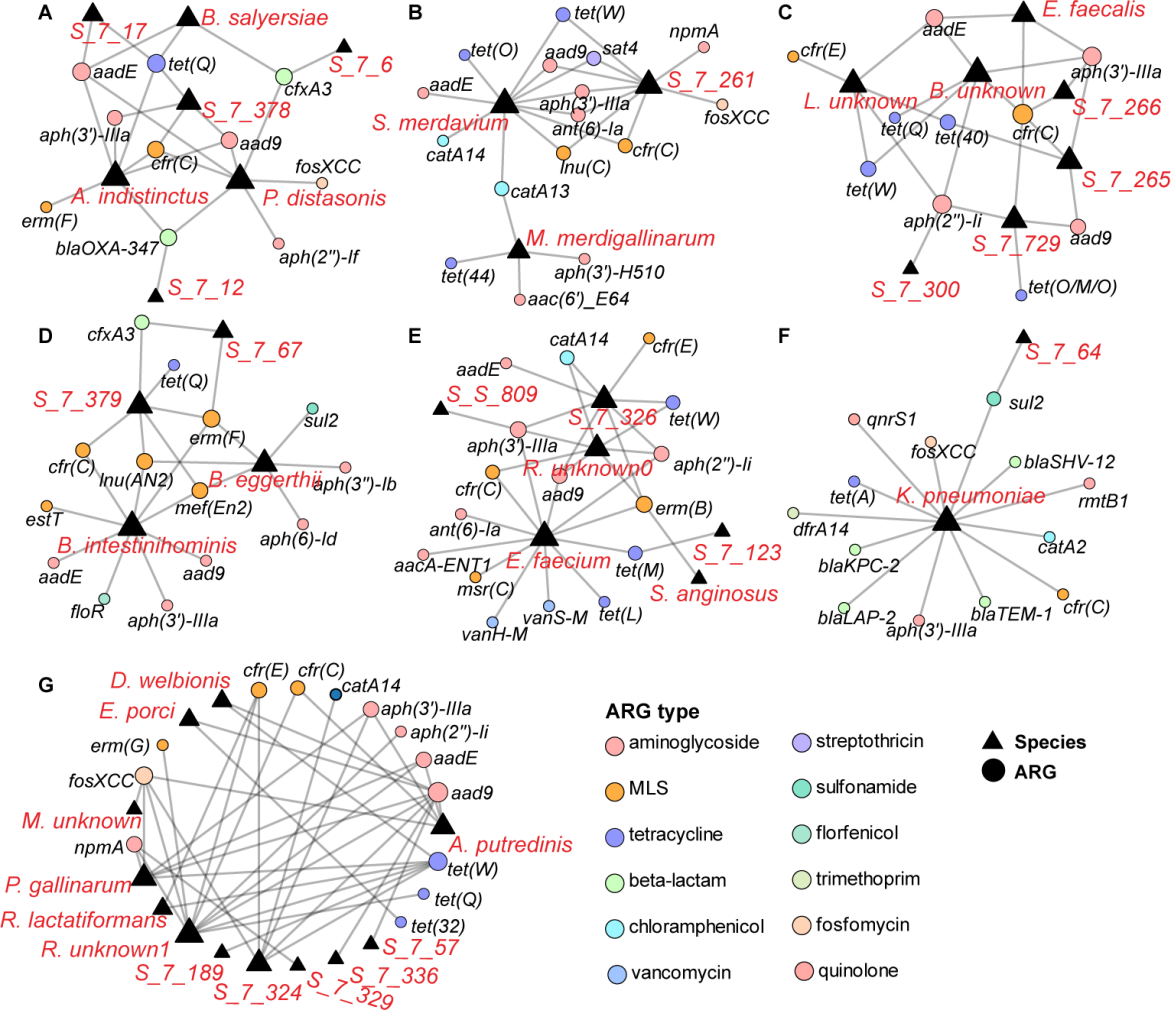
Antibiotic resistance genes (ARGs) in different gut microbiome bacterial strains. This figure illustrates the ARG profiles of seven bacterial strains (**A–G**). Each panel (**A–G**) represents a different bacterial strain. The lines connecting each bacterium and different ARGs represent the specific resistance genes each strain carries. The types of antibiotics to which these genes confer resistance are indicated by different line styles. The ARGs include a variety of resistance genes such as *aph(3’)IIIa, aac(6’)Ie-aph(2’‘)Ia, aad9, tet(O), tet*(M)*, tet(W), cfr/cfr(C), erm(B), erm(F), msr(A), bla_OXA-30_, bla_OXA-347_, bla_OXA-61_, blaTEM1-B1, qnrS1, vanA, dfrA14, floR, catA13, aac(6’)IIc, mph(A), mef(A), aadE-sat4-aadA1, strAB, aadD2, mph(B*), and *fosXCC*. The key on the right side of the figure depicts the color coding for each ARG type. Each panel has a unique set of connections, indicating specific relationships exist between each bacterial species and the ARGs.

Comparing different sets of metagenomic data, we found that the advantage of single-cell analysis lies in its capacity to directly identify bacterial strains harboring specific ARGs, VFGs, and plasmids. Within the human gut microbiome, a total of 13 types of ARGs were identified at the species level of SAGs; these ARGs were found to be carried by bacteria of 41 species, including 22 unknown species and 19 unclassified species (Fig. 2; Tables S3 to S6). Six species in the Bacteroidota bacterial group were commonly found in the human gut—Alistipes *putredinis, Alistipes indistinctus, B. eggerthii, Bacteroides salyersiae, Bacteroides intestinihominis*, and *P. distasonis*, all exhibiting aminoglycoside resistance genes such as *aad*9, *aad*E, and *aph*(2'')-Ii. Notably, certain species were sporadically reported to carry additional genes such as *cfr*(C), *fos*XCC, *bla*_OXA-347_, *tet*(Q), and *cfx*A3. For instance, *B. eggerthii* manifested a diverse repertoire of ARGs, including aminoglycoside, fosfomycin, MLS, sulfonamide, and tetracycline resistance genes, with a total of eleven subtypes of such genes being detected. Similarly, *B. intestinihominis* and *P. distasonis* exhibited a comparable diversity in ARGs.

The Firmicutes group was found to comprise 12 genera and six unknown species. Several taxa were infrequently reported in the human gut, including *M. merdigallinarum, E. porci, S. merdavium, Borkfalkia, Ruthenibacterium*, and *Pararuminococcus gallinarum*. Furthermore, *Enterococcus faecium* strains, which are prevalent in the human gut, were found to harbor five types of ARGs, including those which encoded resistance to aminoglycoside, MLS, tetracycline, vancomycin, and various other agents. These genes could be sub-divided into twelve subtypes, including *aacA-ENT1, aph(2'')-Ii, aph(3')-IIIa, erm(T), fexB, msr(C), poxtA, spw, tet*(L)*, tet*(M), *van*H*-M*, and *van*S-M. Another common bacterium, *S. anginosus*, was found to carry the *erm*(B) gene, which conferred resistance to macrolides and lincosamides.

Uncommon bacteria in the gut, such as *M. merdigallinarum*, *Borkfalkia*, *Eisenbergiella, Ruthenibacterium*, and *Scatomonas*, also exhibited diverse profiles of ARGs. For example, *M. merdigallinarum* was found to harbor genes that encoded resistance to aminoglycosides, chloramphenicol, MLS (macrolide, lincosamide, and streptogramin), and tetracycline. Unknown species within the *Borkfalkia* genus were also found to harbor a range of ARGs, including those that conferred resistance to aminoglycosides, beta-lactams, chloramphenicol, MLS, fosfomycin, and tetracycline. The species *E. porci* was found to exhibit resistance to tetracycline through carriage of *tet*(32) and *tet*(Q). Similarly, the unknown species within the *Ruthenibacterium* genus carried multiple types of ARGs, and a total of nine subtypes of such genes were identified. The species *S. merdavium* exhibited resistance to six types of antibiotics, and as many as fourteen subtypes of ARGs could be detected; these included the *aad*9, *aad*E, *ant* (6)-Ia, *aph*(3')-IIIa, *catA*13, *catA*14, *cfr*(C), *fos*XCC, *lnu*(C), *sat*4, *tet*(O), *tet*(Q), and *tet*(W). At the Firmicutes level, a bacterium within the *Lachnospiraceae* family was found to carry the ARGs of *aad*9, *aph*(2'')-Ii, *cfr*(C), and *tet*(O/M/O).

Among the Proteobacteria, *K. pneumoniae* was found to carry various ARGs, including *bla*_KPC-2_, *bla*_LAP-2_, *catA2*, *dfrA14*, *qnrS1*, *rmtB1*, and *tet(A*). Within the Archaea domain, specifically the phylum Methanobacteriota, *M. smithii* was found not to carry any ARGs. The diversity of ARGs carried by these unclassified bacteria is a hallmark of the complexity of antimicrobial resistance-encoding genetic elements harbored by the microbial communities in the human intestine. These 19 unclassified bacteria collectively harbor 23 subtype ARGs, including *aad9, ant (6)-Ia, aph(2'')-Ii, aph(3')-IIIa, cfr(C), fosXCC, lnu(C), npmA, sat4, sul2, tet*(W), *aadE, bla_OXA-347_, catA14, cfr(E), cfxA3, erm(B), erm(F), lnu(AN2), mef(En2), tet(40), tet*(M), and *tet*(Q).

Analysis of VFGs harbored by the gut microbiome strains showed that only two bacterial strains harbored VFGs at the strain level. *Enterococcus faecium* was found to harbor *fss3, acm*, and *sgrA*, whereas *K. pneumoniae* carried a variety of VFGs including *ybtS, ybtU, ybtT, ybtA, ybtP, ybtS, ybtA, iutA, ompA, ybtQ, ybtS*, and *ybtX*.

Analysis of metagenome-assembled genomes (MAGs) offers insights into the unique genetic features of clinically important bacterial species and their associated ARGs, albeit only partially. In this study, a total of 19 MAGs were identified (Table S7), mostly belonging to Firmicutes, Bacteroidota, and Proteobacteria, with Firmicutes comprising the majority (14 out of 19). However, only 10 MAGs carried ARG genes, with 8 MAGs carrying fewer than 3 ARGs and 2 MAGs carrying 5 ARGs. The predominant types of ARGs identified in MAGs were mainly associated with tetracycline, aminoglycoside, and beta-lactamase resistance. This finding suggests that, although analysis of MAGs can help identify species harboring ARGs, their ability to comprehensively capture ARG data in the gut microbiome is limited.

### Co-evolution of ARGs in different bacterial species

**Fig 3:**
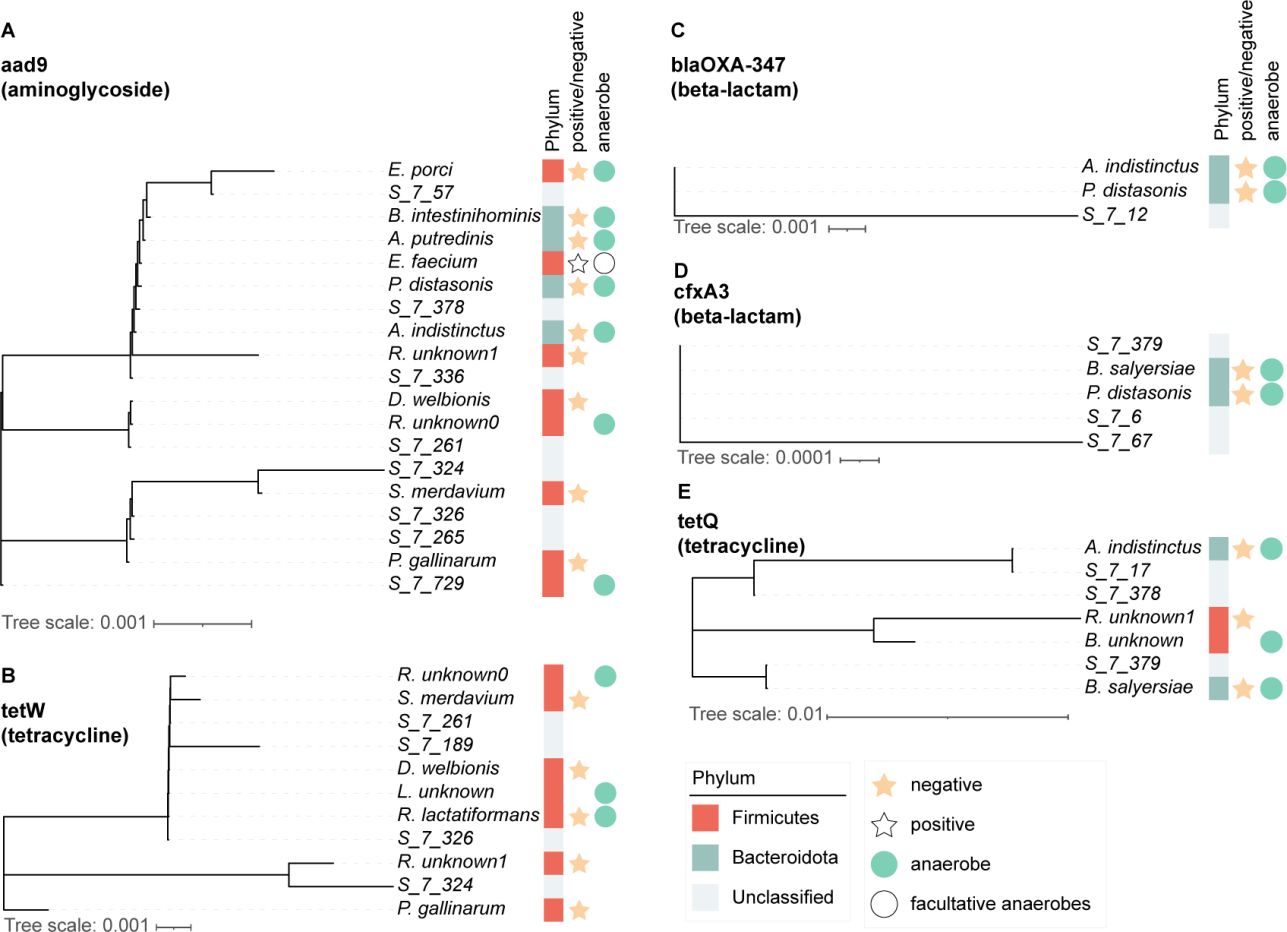
Co-evolution of antibiotic resistance genes (ARGs) in different bacterial species. This figure illustrates the co-evolution features of ARGs in various bacterial species. Each panel (**A–E**) represents a different ARG, with panel A specifically showing the *aad9* gene that is related to aminoglycoside resistance. In panel A, the *aad9* gene is found in 19 different bacterial species, which are further clustered into three distinct groups. The other panels (**B–E**) depict the evolutionary trees of *tet(W), bla_OXA-347_, cfxA3*, and *tet(Q*) genes, which encode tetracycline and beta-lactam antibiotic resistance. The color-coded phyla indicate the diversity of bacteria carrying these ARGs. Additionally, yellow stars indicate gram-negative bacteria, while white stars indicate gram-positive bacteria. This diagram provides a comprehensive overview of the distribution and co-evolution of ARGs among different bacterial species.

In the human gut microbiome, co-evolution of ARGs and transmission between different bacterial species, which primarily involve Bacteroidota, Firmicutes, and unclassified bacteria, is a common event and underscores the dynamic interplay shaping development of microbial resistance in major bacterial pathogens. Notably, the genera such as *Ruthenibacterium, Enterococcus*, and *Alistipes* emerge as key players in the co-evolutionary process. Phylogenetic analysis conducted for ARGs carried by strains of at least three species showed the existence of 8 types of ARGs and that these 8 types comprised a total of 25 sub-types (Fig. 3; Fig. S1 and S2). These subtypes exhibit distinct clustering patterns among various bacterial species, which are indicative of divergent evolutionary trajectories. Within the realm of aminoglycoside resistance genes, a spectrum of subtypes including *AAC(6')-Ie-APH(2'')-Ia*, *AAC6*, *aad (6*), *aad9*, *ant(6)-Ia*, *ANT9*, *aph(2'')-Ii*, *aph(3')-III*, *APH(3')-IIIa*, and *APH2* have been implicated in co-evolutionary dynamics. This co-evolution event predominantly involves representative strains of the Bacteroidota, Firmicutes, and Terrabacteria groups, alongside numerous unidentified bacterial taxa. For instance, the *aad9* gene was found in three discernible clusters across diverse bacterial hosts. Cluster 1 encompasses 13 species, including both anaerobic gram-negative (e.g., *E. porci, B. intestinihominis, A. putredinis, P. distasonis, and A. indistinctus*) and aerobic gram-positive (e.g., *E. faecium and Dysosmobacter welbionis*) bacteria from the Bacteroidota and Firmicutes phyla. Cluster 2 comprises a singular unknown species within the Lachnospiraceae family, andCluster 3 comprises *P. gallinarum, S. merdavium*, and three unidentified species. Similarly, although the *aph(2'')-Ii* gene was not detectable in the metagenomic data, it was found in three distinct clusters involving eight species, including *E. faecium* and various unidentified species within the genera *Borkfalkia, Lawsonibacter, Ruthenibacterium*, and *Lachnospiraceae*.

Co-evolutionary dynamics of the beta-lactamase genes primarily revolve around two elements: *cfxA3* and *bla*_OXA-347_. The *bla*_OXA-347_ gene was detected in three distinct species, namely, *A. indistinctus, P. distasonis*, and species of unknown identity. Conversely, *cfxA3* was mainly found in three discernible clusters: one cluster encompasses *A. indistinctus* and *P. distasonis*, whereas the other two clusters comprise individual unidentified species. Chloramphenicol resistance was encoded by a single gene, *cat*, which was detectable in strains of three distinct subgroups, namely, cluster 1 (Unclassified-7-324; Unclassified-7-326), cluster 2 (*S. merdavium*), and cluster 3 (*Ruthenibacterium*) (Fig. 3; Fig. S1 to S3).

The fosfomycin gene *fos*XCC exhibited a distinct clustering pattern. One cluster encompassed *A. putredinis, K. pneumoniae, P. distasonis*, and *P. gallinarum*, along with two unidentified species of bacteria. Another cluster was associated with the genus *Ruthenibacterium*, while the third cluster involved yet another unidentified bacterial species.

Regarding the MLS genes, which include *cfr(C), cfr(E), erm(F), ErmB, lnu(AN2*), and *mef(En2*), specific clustering patterns emerged. For the *cfr(C*) gene, three distinct clusters were identified: cluster 1 (comprising Unclassified-7-378, *P. distasonis, A. indistinctus*, and *K. pneumoniae*), cluster 2 (including Unclassified-7-265 and Unclassified-7-266), and cluster 3 (featuring *Enterococcus faecalis, Ruthenibacterium* of an unidentified species, *A. putredinis*, Unclassified-7-379, and *B. intestinihominis*). Similarly, for *cfr(E*), clustering revealed the presence of cluster 1 (associated with *Lawsonibacter*), cluster 2 (linked to *R. lactatiformans*), and cluster 3 (comprising Unclassified-7-324, *Ruthenibacterium* of an unidentified species, and Unclassified-7-326). For the *lnu(AN2*) and *mef(En2*) genes, three clusters were identified, all involving the same three species: *K. pneumoniae, B. eggerthii*, and an unidentified species classified as Unclassified-7-64. Moreover, it is noteworthy that the genes *erm(F*) and *ermB* were exclusively detected in single-cell data. For the *erm(F*) gene, clustering revealed the existence of cluster 1 (associated with Unclassified-7-326), cluster 2 (linked to *Ruthenibacterium* of an unidentified species and *S. anginosus*), and cluster 3 (comprising *E. faecium*). As for the *ermB* gene, clustering analysis revealed the presence of cluster 1 (associated with *E. faecium*), cluster 2 (linked to *Ruthenibacterium*), and cluster 3 (involving *S. anginosus* and Unclassified-7-326) (Fig. 3; Fig S1, S2 and S3a through c).

Among the tetracycline genes, particularly *tet*(Q) and *tet*(W), distinct clustering patterns were observed, shedding light on the co-evolution of ARGs within the human gut microbiome. The *tet*(Q) gene, which was exclusively detected in single-cell data, exhibited three clusters: cluster 1 comprised *A. indistinctus*, Unclassified-7-17, and Unclassified-7-378; cluster 2 contained Unclassified-7-379 and *B. salyersiae*; cluster 3 involved *Ruthenibacterium* of an unidentified species and *Borkfalkia* of an unidentified species. Similarly, *tet*(W) could be clustered into three clusters. Cluster 1 was associated with *Ruthenibacterium* of an unidentified species, *S. merdavium*, Unclassified-7-261, Unclassified-7-189, *D. welbionis*, *Lawsonibacter* of an unidentified species, and *Ruthenibacterium lactatiformans*; cluster 2 was linked to *P. gallinarum*; cluster 3 was associated with *Ruthenibacterium* of an unidentified species and Unclassified-7-324. These clustering patterns suggest intricate evolutionary relationships among tetracycline resistance genes and various bacterial species, including unclassified and rare bacteria such as *E. porci, S. merdavium*, and *f_Lachnospiraceae*; s_unknown_0, among others, which were the major players that shaped the ARG landscape within the gut microbiome (Fig. 3; Fig. S3d).

For the *tet*(M) gene, although only two species, *E. faecium* and an unclassified bacteria designated as unclassified bacteria123, carried this gene in the gut microbiome, notable differences in the gene sequence were observed. Specifically, the *tet*(M) gene in unclassified bacteria exhibited a 99.79% similarity to the reference sequence, while the *tet*(M) gene in *E. faecium* displayed a 99.32% similarity, indicating the presence of mutations that involved 19 nucleobase changes and 10 amino acid changes (Fig. 3; Fig. S3e).

### HGT landscape in the gut microbiome

**Fig 4:**
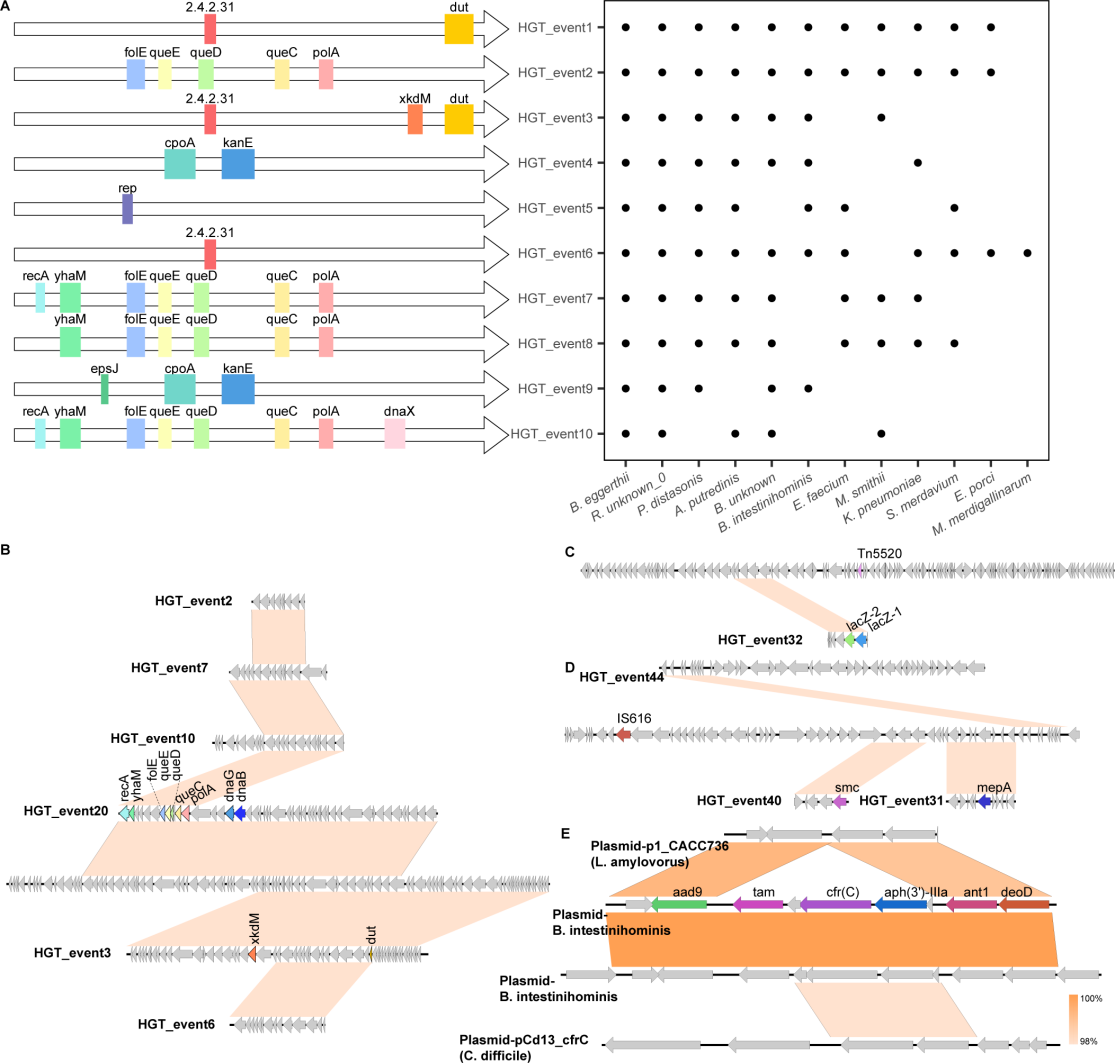
Horizontal Gene Transfer (HGT) events in different bacterial species. This figure illustrates the relationship between HGT events and the bacterial species involved. The left panel shows different genes involved in HGT events, each represented by a colored block and labeled accordingly (e.g., *folE, polA, and queC*). Each row corresponds to a specific HGT event (e.g., HGT_event1 and HGT_event2). The right panel displays a dot plot representing the presence (indicated by black dots) of these specific genes in various bacterial strains (e.g., *E. porci*). (**B**) Shows the largest contigs’ HGT associated with HGT events 2, 7, 10, 20, 3, and 6, carrying the most genes, including *folE*, *polA*, *queC*. **C** and **D** depict the HGT with MGEs. **C** carries Tn5520 and also carries *lacZ-1* and *lacZ-2*, while **D** suggests that HGT events 40 and 44 may have occurred through IS616 and also carries *smc* and *mepA*. **E** shows two plasmid HGT events that occurred in bacteria *B. intestinihominis* and *A. putredinis*, with reference plasmids from *L. amylovorus* and *C. difficile*.

Contigs exceeding five kilobase pairs were chosen to study HGT events that occurred in Firmicutes, Bacteroidota, and Proteobacteria, with one case involving Archaea from the Methanobacteriota phylum. A total of 309 HGT events involving 14 species were identified; this phenomenon suggested that extensive genetic exchange occurred within the human gut microbiome (Fig. 4; Table S8).

HGT1, the most prevalent HGT event, was observed 28 times. This event involved the transfer of the gene *dut* (deoxyuridine triphosphatase) and the gene that encoded ADP-ribosyltransferase (EC 2.4.2.31), among other hypothetical protein genes. This event was observed in 11 bacterial species, including *A. putredinis, B. eggerthii, B. intestinihominis, Borkfalkia unknown_0, E. porci, E. faecium, K. pneumoniae, M. smithii, P. distasonis, Ruthenibacterium unknown_0*, and *S. merdavium*. HGT3 and HGT11 shared similarities with HGT1, with HGT3 involving the transfer of only the *dut* gene, while HGT11 involved an additional gene, *xkdM*. The *dut* gene is essential for DNA replication and repair in bacteria. These HGT events indicate the exchange of genetic material involved in crucial cellular functions among diverse bacterial species within the gut microbiome.

HGT2, the second-most prevalent HGT event, involved the transfer of the genes *folE, polA, queC, queD*, and *queE* among 11 bacterial species. This event excluded *M. merdigallinarum, S. anginosus*, and one unidentified species within the Lachnospiraceae family. Other HGT events, such as HGT7 and HGT10, were similar to HGT2 but included additional genes. HGT7 involved transfer of the genes *recA* and *yhaM*, while HGT10 incorporated even more genes, including *dnaX, recA*, and *yhaM*. The presence of DNA repair and replication genes like *polA, recA*, and *dnaX* in these HGT events suggests the potential enhancement of cellular mechanisms related to DNA maintenance and genomic stability in the recipient bacteria. Improved DNA repair and replication capabilities can contribute to the overall fitness and survival of bacteria, especially under prolonged treatment with antibiotics or other environmental stresses.

HGT4 involved transfer of the gene pair *cpoA* and *kanE*, along with other hypothetical protein genes, among six bacterial species: *A. putredinis, Barnesiella intestinihominis, B. eggerthii*, an unidentified species of *Borkfalkia, K. pneumoniae*, an unidentified species of *Ruthenibacterium*, and *P. distasonis*. Similarly, HGT9 shared similarities with HGT4 but included an additional gene, *epsJ*. On the other hand, HGT5 was characterized by the presence of a single gene named *rep*, which was often accompanied by other hypothetical protein genes. This HGT event was observable in seven bacterial species: *A. putredinis, B. eggerthii, B. intestinihominis, E. faecium, P. distasonis*, an unidentified species of *Ruthenibacterium*, and *S. merdavium*.

Furthermore, there were 54 other HGT events, each occurring fewer than 10 times between different species, which involved functionally important genes. Despite their lower frequency, these events were still regarded as being significant in terms of enhancing bacterial survival fitness under adverse environmental conditions. For instance, in HGT 25, genes such as *baeS, saeR, bceB*, and *yxdL* were identified. These genes have been observed to undergo HGT across different bacterial species, contributing to cellular responses to environmental stress, biofilm formation, antibiotic sensing and resistance, and functions of the ABC transport systems. Another notable example is HGT 35, which involved the genes *ant1, cfr, deoD, bioC, aadK*, and *aphA*. This particular HGT event was mediated by plasmids harbored by *B. intestinihominis* and *A. putredinis*, indicating that transmission of resistance genes through this event is common. It is worth mentioning that *P. distasonis* exhibited 76 HGT events, while *E. porci* had 52, and *A. putredinis* had 38 events. Additionally, *Acrea M. smithii* participated in 11 HGT events, along with *K. pneumoniae, S. merdavium, B. eggerthii*, an unidentified species of *Borkfalkia*, and an unidentified species of *Ruthenibacterium*.

### Strain-level characterization of *K. pneumoniae*

**Fig 5:**
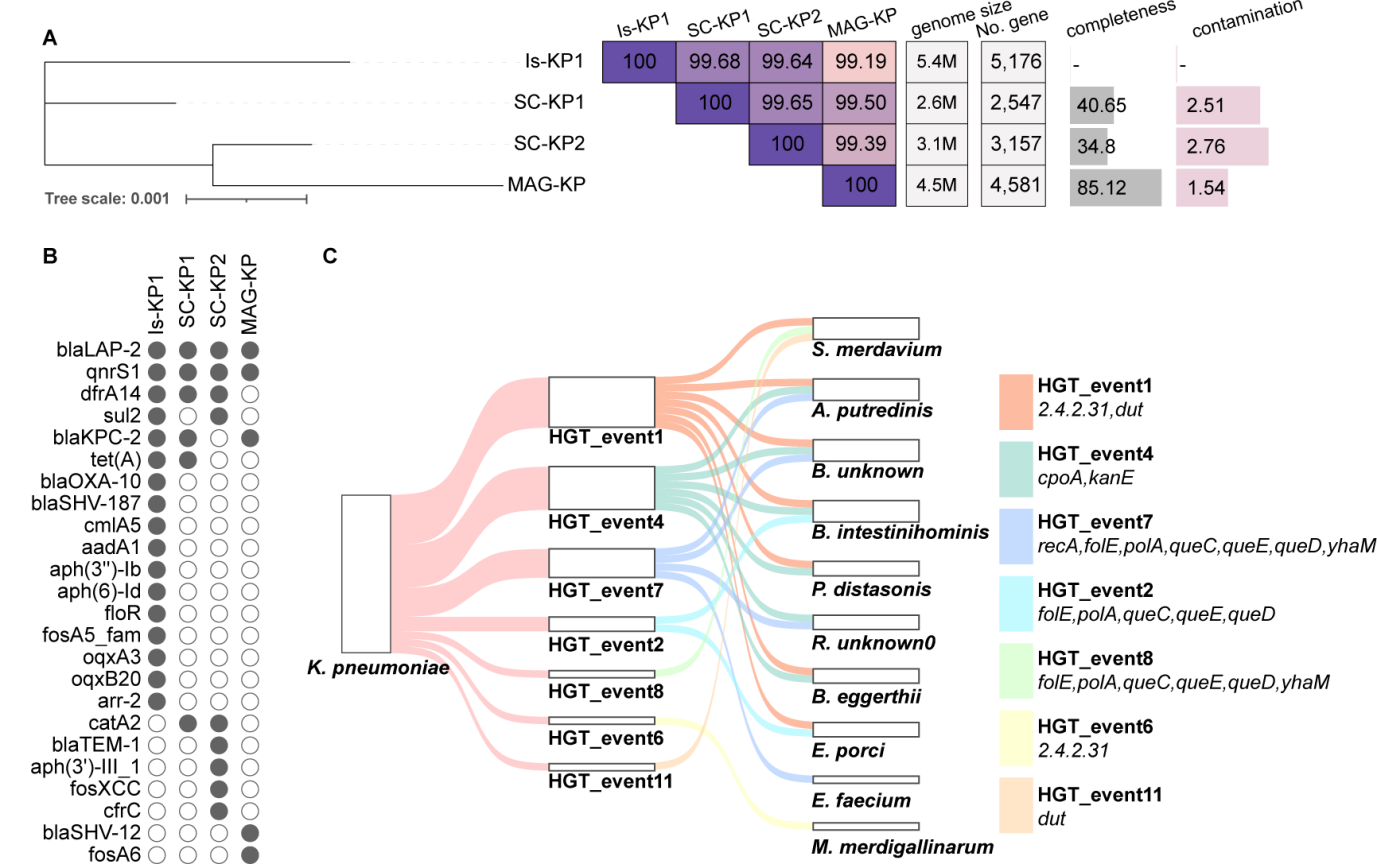
Phylogenetic analysis, antibiotic resistance genes (ARGs), and horizontal gene transfer (HGT) events in major gut microbiome bacterial strains. **A** displays a phylogenetic tree representing the evolutionary relationships among different bacterial strains, along with ANI comparison values, genome sizes, gene completeness, and contamination levels. **B** illustrates the ARGs each strain carries. **C** shows the HGT events that occurred between *K. pneumoniae* (KP) and other bacterial species.

The IS-KP1 strain isolated from the patient’s gut exhibited a concerning array of ARGs, indicating that this strain possessed high-level resistance to various antibiotics commonly used in clinical settings. These ARGs included *aadA1, aph(3'')-Ib, aph (6)-Id, arr-2, bla_KPC-2_, bla_LAP-2_, bla_OXA-10_, bla_SHV-187_, cmlA5, dfrA14, floR, fosA5_fam, oqxA3, oqxB20, qnrS1, sul2*, and *tet(A)* (Fig. 5; Table S10). Each of these genes confers resistance to specific classes of antibiotics, suggesting that the IS-KP1 strain was multidrug- resistant. Moreover, the presence of several VFGs in the IS-KP1 strain indicates that this strain also exhibited high-level virulence. The VFGs, including *iucA, iucB*, and *ecpR*, are known to play crucial roles in several virulence mechanisms in major bacterial pathogens.

The utilization of single-cell analysis allowed us to discover two distinct strains of *K. pneumoniae*, demonstrating that this method is highly efficient in delineating strain-level characteristics of clinically relevant pathogens. Furthermore, metagenomic analysis led to the identification of one MAG that could be assigned to *K. pneumoniae* (denoted as MAG-KP1). Phylogenetic tree construction and average nucleotide identity (ANI) comparison revealed that one strain, SC-KP1, exhibited a high degree of similarity to the isolate IS-KP1, with a 99.56% ANI. Conversely, the other strain, SC-KP2, displayed a slightly lower similarity, with a 99.51% ANI to IS-KP1. However, MAG-KP1 showed a lower similarity, with a 99.19% ANI to IS-KP1, despite having a higher completeness level at 85%. Interestingly, SC-KP1 harbored only six ARG genes, which was the same as that of IS-KP1. In contrast, SC-KP2 carried nine ARGs, including three genes (*fosXCC, cfrC, and aph(3')-III_1*), which were absent in IS-KP1.

The finding that *K. pneumoniae* was involved in numerous ARG co-evolution events underscores its role in shaping the resistance landscape within the gut microbiome. Several key ARGs, including *APH(3')-IIIa, cfr(C), FosXCC*, and *sul2*, have exhibited notable co-evolution patterns. *APH*(3')-IIIa was linked with a diverse range of bacteria including *P. gallinarum, S. merdavium, Enterococcus faecalis, B. intestinihominis, A. putredinis, E. faecium, A. indistinctus*, and various unknown species. These associations formed three distinct clusters, highlighting the diversity and interconnectedness of ARGs across different bacterial taxa. Similarly, *cfr(C*) was found to have evolved into three clusters, involving bacteria such as *P. distasonis, A. indistinctus, K. pneumoniae, Enterococcus faecalis, B. intestinihominis*, and *Ruthenibacterium* unknown-1. Notably, certain strains of unknown bacterial species formed unique clusters, indicating that such strains also exhibited evolutionary trajectories in ARG acquisition and dissemination. Furthermore, evidence of evolution of the *fos*XCC gene was obtained in bacteria such as *A. putredinis, K. pneumoniae, P. distasonis*, an unknown species in *Massilistercora, P. gallinarum, Ruthenibacterium* unknown-1, and an unclassified group. These findings showed that the *fos*XCC gene could be found in bacteria of diverse genetic backgrounds. The *sul2* gene was identified in *K. pneumoniae, B. eggerthii*, and an unknown species (Unclassified-7-64), indicating its presence across different bacterial taxa. Importantly, these ARGs were commonly associated with SC-KP2, suggesting that they play a crucial role in conferring phenotypic antibiotic resistance in this strain.

In the gut microbiome analyzed in this work, *K. pneumoniae* was found to actively engage in HGT with 11 bacterial species. One notable event, namely, HGT2 as mentioned above, involved seven species, including *B. eggerthii, Borkfalkia unknown_0, Ruthenibacterium unknown_0, S. merdavium, E. faecium, A. putredinis, and B. intestinihominis*. This event involved the transfer of genes such as *folE, polA, queC, queD*, and *queE*, which encoded various physiological functions such as folate biosynthesis, DNA polymerization, and quorum sensing. Another significant HGT event involved the *dut* gene and was found to involve eight bacterial species: *B. eggerthii, Borkfalkia unknown_0, S. merdavium, E. porci, M. merdigallinarum, P. distasonis, A. putredinis*, and *B. intestinihominis*. In addition, an HGT event involves transfer of the genes *cpoA* and *kanE* among *B. eggerthii, Borkfalkia unknown_0, Ruthenibacterium unknown_0, P. distasonis, A. putredinis, B. intestinihominis*, and *K. pneumoniae*.

## DISCUSSION

Single-cell analysis of the patient’s gut microbiome provides a powerful means to investigate the interaction between pathogenic and commensal bacteria during infection and antibiotic treatment. In this study, the patient was initially treated with cefuroxime sodium, followed by meropenem and linezolid as symptoms evolved. This sequential antibiotic regimen illustrates the adaptive clinical management required to control infection. Through single-cell analysis, we aimed to uncover microbiome composition shifts, explore resistance mechanisms, and assess treatment effectiveness.

The single-cell analysis in this study identified a diverse range of bacterial and a few archaeal genomes in the gut microbiome of the patient. Compared with a previous single-cell study of the normal gut microbiome that reported 100 bacterial species (19), our analysis revealed 11 species, nine of which were genera exclusively found in this patient. Notably, *K. pneumoniae* and *Enterococcus faecium*, both recognized as pathogens, were among the identified species. Species such as *M. merdigallinarum, Borkfalkia, E. porci, S. merdavium*, and *M. smithii* are infrequently reported in the human gut, and their genomes were rarely documented. However, such organisms could be identified in the single-cell SAGs in this work when compared to the metagenomic data. These species were also found to be predominantly involved in the evolution of ARGs and HGT events. In addition, a substantial number of unclassified and unknown bacteria were also detected, and these organisms also contributed to ARG evolution and HGT. Following antimicrobial treatment, the microbiome appeared to have undergone significant changes in the composition, favoring the proliferation of resistant bacteria while suppressing sensitive strains. Single-cell technology proved invaluable in detecting these pathogens, as well as uncommon and unclassified bacterial species, showcasing its application potential in clinical research.

Single-cell analysis offers the opportunity to directly detect ARGs harbored by specific bacterial strains in the human gut microbiome. Such capability is unmatched by other methods such as metagenomics analysis. Notably, this study revealed the presence of rare bacteria carrying ARGs, such as *M. merdigallinarum,* which harbored *cfr*(C), a gene typically associated with coagulase-negative *Staphylococci, Staphylococcus aureus*, and Enterobacteriaceae (20). This study uncovered 25 ARG subtypes, including *tet*(Q) and *cfr*(C), in multiple clusters. Notably, some genes like *aph(2'')-Ii, ErmB*, and *tet*(Q) were not detectable in metagenomic data, indicating that the resolution of single-cell analysis was much higher. Our data showed that uncommon and unknown bacteria played significant roles in ARG evolution. Examples include *S. merdavium,* which carried 9 ARG subtypes, and *Ruthenibacterium,* which harbored 15 ARG subtypes. In addition, the ARG subtypes of strains of these species often exhibited mutations when compared to the reference genes. Single-cell analysis not only allowed detection of specific or uncommon bacteria but also provided significant insights into their involvement in ARG evolution, which was not achievable by conventional metagenomic methods. Moreover, single-cell analysis allows direct detection of VFGs and plasmids harbored by specific bacterial strains within the human gut microbiome.

Our analysis highlights the involvement of 14 bacterial species in HGT events, with noteworthy contributions from less-studied taxa like *E. porci, S. anginosus*, and *M. merdigallinarum*. Our data, therefore, showed that bacterial strains of broad taxonomic diversity participated in genetic exchange within the gut microbiome. Specific genes like *folE, polA, queC, queD*, and *queE* were implicated in these HGT events, suggesting that acquisition of these genes is a key step in antibiotic resistance development in various gut microbiome strains and that these events contributed to the dissemination of functional traits among bacterial populations. Distinct clustering patterns of HGT events among different species indicate varying preferences for genetic exchange and existence of ecological niches that underlie the HGT dynamics. Notably, genes associated with plasmid transfer, such as *mbeA, mbeC*, and *relG*, exhibited specific associations with certain bacterial species, emphasizing the role of mobile genetic elements in facilitating HGT within microbial communities. Compared to a healthy human gut microbiome (19) using single-cell sequencing, our study reveals differences in HGT dynamics. Specific genes like *traC, comB2, topB, radC*, and *wbpO* were found to be prominent in healthy human cases. However, the main HGT genes identified in this study were *folE, polA, queC, queE, queD*, and *dut*, involving 13 bacterial species. These genes play a crucial role in enhancing the ability of the recipient strains to repair DNA damage and maintain genomic stability, which contributes to their overall fitness, especially after prolonged treatment with antibiotics.

Comparison between isolated genomes, such as that of the strain IS-KP1 and other single-cell genomes, offers detailed insights into the co-evolutionary dynamics and HGT mechanisms of *K. pneumoniae* within the human gut microbiome. Isolated genomes of *K. pneumoniae* exhibited a diverse array of ARGs and VFGs, confirming that this species is a clinically important pathogen. On the other hand, single-cell analysis distinguished two distinct *K. pneumoniae* strains, SC-KP1 and SC-KP2, each with unique genomic features. While SC-KP1 shares a similar ARG profile with IS-KP1, SC-KP2 carries additional ARGs, highlighting strain-specific differences in resistance. The co-evolution of these strains within the gut microbiome is reflected in the ARG transfer events involving key genes such as *APH(3')-IIIa*, *cfr(C*), *FosXCC*, and *sul2*, which are predominantly found in SC-KP2. These genes were found to be broadly distributed across diverse bacterial species, including the less explored gut inhabitants such as *P. distasonis* and *P. gallinarum*, highlighting the complex ecological dynamics in the gut microbiome that drive resistance gene dissemination and bacterial evolution. In addition, studies of HGT events between *K. pneumoniae* and other gut-associated species reveal the importance of genetic exchange in shaping bacterial adaptation to environmental changes and pathogenicity. Notable HGT events, such as those involving genes like *cpoA* and *kanE*, exhibit the potential of conferring antimicrobial resistance and enhancing bacterial fitness.

However, it is important to note that these findings are derived from a single patient, which limits the ability to generalize the results to broader populations. The gut microbiome is highly individual-specific and shaped by numerous factors, including genetics, diet, health status, and treatment history. Therefore, the microbial dynamics, ARG profiles, and HGT patterns observed in this study may not be representative of those in other individuals or patient groups. While this study provides a high-resolution view of microbial adaptation, it is inherently limited by its focus on a single patient. The findings, including the extensive HGT events and the micro-diversity of resistance strains, are illustrative of the processes that can occur within a complex gut ecosystem under strong selective pressure. However, individual factors such as the host’s unique native microbiota, genetics, and exact medical history undoubtedly influence these dynamics. Therefore, the generalizability of these specific results may be limited. Future studies with larger patient cohorts, stratified by disease state and treatment regimen, will be essential to confirm the prevalence and patterns of the adaptation mechanisms reported here. This work serves as a critical proof of concept, demonstrating the power of single-cell genomics to uncover these complex within-host evolutionary dynamics.

This study has several limitations. First, the analysis was confined to the gut microbiome. While the patient was treated for a suspected pulmonary infection, no corresponding respiratory samples were available for culture or sequencing. Therefore, we cannot establish a direct link between the resistant pathogens found in the gut and the initial cause of the lung inflammation. However, our findings provide a detailed map of the ARG landscape that emerged in the gut as a consequence of therapy, highlighting its role as a potential reservoir for resistance. The resistance genes identified in the gut microbiome, such as *bla*_OXA-347_, likely originated from environmental exposure—particularly the hospital setting—which serves as a common reservoir for antibiotic-resistant bacteria. These genes were probably acquired by the patient and subsequently established within the gut microbial community. Under the potent selective pressure of antimicrobial treatment, these resistance determinants were enriched and maintained, potentially through HGT to other commensal bacteria, allowing them to persist and evolve as a functional component of the patient’s adaptive gut resistome. The detection of high-risk resistance genes like *bla*_OXA-347_ likely reflects initial acquisition from the hospital environment, where cross-colonization with multidrug-resistant organisms is common. However, our single-cell data demonstrate these genes were not merely transient contaminants but were functionally established within the gut microbiome. The presence of these genes within multiple Single Amplified Genomes (SAGs), evidence of *in situ* mutation, and widespread HGT confirm they underwent active selection and evolution under antibiotic pressure, transforming the gut into a reservoir of adapted resistance.

While the resistance phenotype of the isolated *K. pneumoniae* strain was confirmed through antimicrobial susceptibility testing, the functional expression of resistance genes identified via single-cell sequencing in uncultured taxa warrants further investigation. Future studies should prioritize developing methods for phenotypic validation—such as heterologous expression or advanced culturing techniques—to directly link these genotypic profiles to observable resistance traits in diverse gut microbiota.

Future studies involving larger and more diverse cohorts are needed to validate these observations and to further elucidate the roles of rare and uncharacterized bacterial species in antimicrobial resistance evolution and pathogen ecology. Integrating single-cell approaches with longitudinal sampling and complementary techniques will also be critical for expanding our understanding of microbiome dynamics in clinical settings.

### Conclusion

In this study, single-cell analysis was found to be highly effective in unraveling the intricate dynamics of bacterial colonization, antibiotic resistance development, and HGT events within the human gut microbiome. Through this approach, we identified a diverse array of bacterial species, including uncommon taxa rarely found in human gut microbiome, and directly detected ARGs and VFGs harbored by specific strains. The study revealed a complex landscape of ARG evolution that involved both common and uncommon bacteria and highlighted the role of HGT in disseminating functional traits across bacterial populations. Notably, specific genes and gene clusters implicated in HGT events were identified, shedding light on their diverse roles in bacterial adaptation to changes in environmental conditions and niche specialization. By juxtaposing isolated genomes with single-cell genomes, we gained insights into the co-evolutionary dynamics and strain-specific variations in antimicrobial resistance development, particularly in pathogens like *K. pneumoniae*. Despite the limitations of single-cell analysis, including relatively low sample representation, our findings underscore its significant potential in advancing our understanding of the ecology of the human gut microbiome and facilitating development of strategies for combating antibiotic resistance and infectious diseases in clinical settings.

## MATERIALS AND METHODS

### Patient case

An adult male was admitted on 18 November 2023 with "acute cerebral hemorrhage." Cefuroxime sodium was given on Day 2. Treatment changed to meropenem and linezolid on Day 11 due to fever and high inflammation. Linezolid was stopped on Day 19. By Day 21, the patient had diarrhea and constipation; rectal swabs showed carbapenemase-producing *K. pneumoniae*. Meropenem was stopped on Day 27 after inflammation normalized. The patient left the ICU on Day 31. A rectal sample for single-cell analysis was taken on 12 December 2023. See supplementary methods for details.

### Isolation and lysis

The specimens were mixed with 25% glycerin and frozen at −80°C. For each experiment, 1-3 μL aliquots were incubated at 37°C for 30 minutes, centrifuged at 13,400 rpm for 1 minute, and 900 µL of the supernatant was discarded. This was repeated three times with 900 µL of PBS. The final bacterial concentration was adjusted to 50 × 10^^6^ cells/mL and then mixed with OptiPrep. A lysis reagent mix (240 µL) included green buffer, lysozyme, prepgem, lysostaphin, BSA, random hexamer, and water. The microbial suspension was connected to a device via a syringe and polyethylene tubing, with flow rates set for microbial suspension, lysis reagents, and oil. Droplets were collected and transferred to a PCR tube, with mineral oil added to prevent evaporation. The lysis program involved incubation at 37°C for 30 minutes, 75°C for 15 minutes, and 95°C for 5 minutes, followed by storage at 4°C. See supplementary methods for details.

### Whole-genome amplification

In whole-genome amplification, a 100 µL MDA mix included phi29 DNA Polymerase Buffer, random hexamers, dNTPs, phi29 DNA Polymerase, BSA, Tween-20, and T2, adjusted with water. Droplet emulsion was injected into an M1 device, paired with droplets containing MDA reagents. Electric fields merged droplet pairs, initiating genome amplification. Incubation at 30°C for 8 hours, followed by 65°C for 10 minutes, was conducted, with samples stored at 4°C for analysis. See supplementary methods for details.

### Droplet-Based tagmentation and PCR barcoding in DNA sequencing library preparation

In the experimental workflow, tagmentation involved preparing a 90 µL Nextera mix with Tagment Buffer L, Tagment DNA Enzymes B and C, BSA, and water. Droplets containing sample and tagmentation reagents were merged using the M1 device, followed by incubation at 55°C for 10 minutes and storage at 10°C. For PCR barcoding, a 420 µL PCR mix was prepared with water, reverse primer, BSA, and Tween-20. Sample droplets were merged with PCR reagents and barcoding beads using the M1 device. After UV exposure to release bar code oligos, PCR included denaturation, annealing, and extension cycles, with final storage at 4°C. Following purification with PFO and AMPure beads, DNA samples were pre-amplified using a mix of pre-amplification reagents. Post-purification steps involved ethanol washing and resuspension in water. Fragmentation and linking were performed to prepare samples for sequencing. See supplementary methods for further details.

### Purification and amplification strategy for library fragment

After adding 69 µL of NF-H2O and 0.3X AMPure beads to the amplification product, incubate the mixture at room temperature for 5 minutes. Transfer the supernatant to a new centrifuge tube after placing it on a magnetic rack for 5 minutes. Add 0.3X AMPure beads to the tube and incubate again at room temperature for 5 minutes. Discard the supernatant after placing the tube on a magnetic rack for 5 minutes. Clean the AMPure beads twice with 1 mL of freshly prepared 80% ethanol. Allow the beads to dry, add 22 µL of ultrapure water, and incubate at room temperature for 5 minutes. Transfer 20 µL of the sample to a 200 µL centrifuge tube. For library tag amplification, prepare a 30 µL mix with 25 µL of amplification mixes, 2.5 µL of amplification primer 1 (Illumina S50X), and 2.5 µL of amplification primer 2 (Illumina N70X). Mix with the sample, and incubate at 98°C for 30 seconds, followed by 11 cycles of denaturation at 98°C for 10 seconds, annealing/extension at 65°C for 75 seconds, and a final extension at 65°C for 5 minutes. Store samples at 4°C for further analysis. Following amplification, add 50 µL of NF-H2O to the product and introduce 0.5X AMPure beads. Mix thoroughly, and incubate at room temperature for 5 minutes. Transfer the supernatant to a new tube after placing it on a magnetic rack for 5 minutes. Add 0.3X AMPure beads to the tube with the supernatant, mix thoroughly, and incubate at room temperature for 5 minutes. Discard the supernatant after placing the tube on a magnetic rack for 5 minutes. Clean the AMPure beads twice with 1 mL of freshly prepared 80% ethanol. Allow the beads to dry, add 22 µL of ultra-pure water, and incubate at room temperature for 5 minutes. Transfer 20 µL of the sample to a 200 µL centrifuge tube. See supplementary methods for further details.

### Genomic analysis pipeline for single amplified genomes (SAGs)

Raw sequencing data underwent initial preprocessing with Fastp v0.24.1 (21) and Cutadapt v5.0 (22) to remove adapters and low-quality sequences. Barcoded reads were then segregated into separate files using a custom Python script. SAGs were individually *de novo-*assembled using SPAdes v4.2.0 (23), followed by quality assessment using CheckM v1.2.3 (24) and genome comparison via Sourmash v4.9.0 (25). Hierarchical clustering grouped SAGs into bins based on genome similarities. Bins were refined iteratively, combining genomes exceeding 95% ANI and filtering contigs based on the length and coverage. Genomes meeting quality thresholds were taxonomically classified using GTDB-Tk 2.4.1 (26). See supplementary methods for further details.

### Phylogeny analysis of genomes

The phylogeny of high- or medium-quality bins was constructed using Anvi'o. Amino acid sequences of six ribosomal proteins (Ribosomal_L1, Ribosomal_L2, Ribosomal_L3, Ribosomal_L4, Ribosomal_L5, and Ribosomal_L6) were extracted, concatenated, and utilized in the analysis. The resulting phylogenomic tree was visualized using ggtree V3.16.0 (27).

### Differentiating strains of the same species

Using Bcftools V1.21 (28), SNPs were identified in high- and medium-quality genomes. Hierarchical clustering based on SNP vectors revealed clusters of SAGs sharing similar SNP profiles, indicating strain proximity. UMAP V0.5.8 (29) plots further visualized SNP data dimensionally. Consensus genotyping of strains involved comparing SNP vectors, excluding ambiguous SAGs. Finally, strain-resolved genomes were co-assembled using SPAdes from SAG reads assigned to each strain. See supplementary methods for further details.

### Horizontal gene transfer analysis

To detect HGT events, we identified DNA blocks > 5,000 bp with > 99.98% identity using blastp. SAGs were filtered based on read alignment ratios (>90%) to reduce contamination. Validation involved examining SAG reads covering HGT sequences between species A and B. Statistical modeling assessed the likelihood of contamination, assuming a 20% worst-case contamination rate. Gene prediction on HGT sequences was performed using prokka (version 1.14.5) (30). See supplementary methods for further details.

### Sequencing of metagenomic DNA

Metagenomic sequencing was applied to the same sample used for single-cell sequencing (sampled on 12 December 2023) to validate DNA consistency and minimize batch effects. The genomic DNA was fragmented using the Frag enzyme, resulting in 350 bp-sized fragments. These fragments underwent end-polishing, A-tailing, and adapter ligation for DNB sequencing, followed by PCR amplification. The PCR product was heat-denatured with a complementary molecule and then ligated using DNA ligase. The remaining linear molecule was digested with exonuclease, producing a single-strand circular DNA library. Library quality was assessed using Qubit for quantification, real-time PCR, and a bioanalyzer for size distribution analysis. Quantified libraries were evenly pooled to form DNA Nanoballs (DNB), which were subsequently sequenced on a DNBseq-T7 platform with a PE150 read length, yielding 20 G of raw data per sample.

### Assembly of metagenomes, prediction of genes, taxonomic assignments, and statistical analysis

Raw sequences underwent initial preprocessing with Trimmomatic (v0.39) to remove adapters, reads < 36 bases, and those with quality scores < 15. KneadData (v0.7.7) eliminated host contamination. Cleaned reads were aligned to microbial marker genes using Bowtie2 (v2.5.1) via MetaPhlAn4. Assembly used megahit v1.2.9; metaSPAdes assembled NGS reads. Binning employed Maxbin 2.0, MetaBAT2, and CONCOCT within metaWRAP v1.3, refined with bin_refinement. MAG quality was assessed by CheckM, assigned to SGBs by GTDB-Tk, and phylogenetic trees constructed with PhyloPhlAn 3.0 in iTOL. See supplementary methods for further details.

## Data Availability

The data have been submitted to NCBI under BioProject accession number PRJNA1111288.

## SUPPLEMENTAL MATERIAL

Supplemental material msystems.01631-24-s0001.docx Supplemental text and Fig. S1-S3.

Supplemental tables msystems.01631-24-s0002.xlsx Tables S1-S10.

ASM does not own the copyrights to Supplemental Material that may be linked to, or accessed through, an article. The authors have granted ASM a non-exclusive, world-wide license to publish the Supplemental Material files. Please contact the corresponding author directly for reuse.
