# Supplementary material for: Elucidation of population-based bacterial adaptation to antimicrobial treatment by single-cell sequencing analysis of the gut microbiome of a hospital patient
Source: mSystems. 2025 Dec 30;11(2):e01631-24. doi: 10.1128/msystems.01631-24 (PMC12911351; doi:10.1128/msystems.01631-24)
Supplement: Supplemental material — Supplemental text and Fig. S1-S3. [file msystems.01631-24-s0001.docx]

**Elucidation of Population-Based Bacterial Adaptation to Antimicrobial Treatment by Single-Cell Sequencing Analysis of Gut Microbiome of a Hospital Patient**

Lianwei Ye^1,2,3#^, Yuchen Wu^1#^, Jiubiao Guo^5#^, Hanyu Wang^1^, Jing Cai^6^, Kaichao Chen^1,3^, Ning Dong^7^, Jiale Yu^8^, Shan Chao^8^, Hongwei Zhou^1^, Gongxiang Chen^1^, Rong Zhang^1*^, Sheng Chen^3,4*^.

1 Department of Clinical Laboratory, Second Affiliated Hospital of Zhejiang University, School of Medicine, Hangzhou, China

2 Department of Infectious Diseases and Public Health, Jockey Club College of Veterinary Medicine and Life Sciences, City University of Hong Kong, Kowloon, Hong Kong

3 State Key Laboratory of Chemical Biology and Drug Discovery and the Department of Food Science and Nutrition, The Hong Kong Polytechnic University

4 Shenzhen Key Laboratory for Food Biological Safety Control, The Hong Kong Polytechnic University Shenzhen Research Institute, Shenzhen, China.

5 Clinical Research Center, The First Affiliated Hospital of Shantou University Medical College, Shantou, China

6 Neuroscience intensive care unit, Second Affiliated Hospital of Zhejiang University, School of Medicine, Hangzhou, China.

7 Center for Clinical Big Data and Analytics, Second Affiliated Hospital and School of Public Health, Zhejiang University School of Medicine, Hangzhou, China

8 MobiDrop (Zhejiang) Co., Ltd

# These authors contributed equally to this work and share first authorship

*Corresponding authors: Sheng Chen, Tel: 852 3400 8619; Rong Zhang, Tel:+8613906535410; Email: [sheng.chen@polyu.edu.hk](mailto:sheng.chen@polyu.edu.hk) ; [zhang-rong@zju.edu.cn](mailto:zhang-rong@zju.edu.cn)

**Methods**

**Patient case**

An adult male patient was admitted to our hospital on November 18, 2023, presenting with symptoms indicative of "acute cerebral hemorrhage." Initial diagnostic imaging via CT scan revealed bilateral pulmonary inflammation alongside a minor pleural effusion on the right side. Subsequently, on the second day of admission, intravenous administration of cefuroxime sodium was initiated to mitigate potential infectious complications. However, by November 28, 2023 (Day 11), the patient's clinical condition deteriorated, marked by the onset of fever, productive cough, and elevated inflammatory biomarkers. In response to these exacerbations, the therapeutic regimen was modified to include intravenous meropenem and linezolid, aimed at broad-spectrum coverage against both Gram-positive and Gram-negative pathogens. By December 6, 2023 (Day 19), observable clinical improvement prompted the discontinuation of linezolid therapy. Nonetheless, on December 8, 2023 (Day 21), the patient exhibited gastrointestinal disturbances, manifesting as alternating diarrhea and constipation. Subsequent rectal swab culturing revealed the presence of carbapenemase-producing Klebsiella pneumoniae subsp. pneumoniae (kpn) strains within the patient's intestinal microbiota. A follow-up rectal swab collected on December 11, 2023 (Day 24), corroborated these findings. Remarkably, no Klebsiella pneumoniae was isolated from respiratory tract specimens. On December 14, 2023 (Day 27), the patient's inflammatory markers normalized, prompting the cessation of meropenem therapy. Consequently, on December 18, 2023 (Day 31), the patient's stabilized cerebral hemorrhage warranted transfer out of the intensive care unit (ICU) to the rehabilitation department for ongoing management. Furthermore, rectal sample acquisition for single-cell analysis was conducted on December 12, 2023, to complement the clinical data with a molecular understanding of the gut microbiota dynamics in the context of infection and treatment response.

**Isolation and lysis**

The specimens were prepared by admixing them with 25% glycerin and subsequently subjected to long-term freezing at -80°C. Each experimental iteration involved the extraction of 1-3 μL aliquots of specimens, which were then incubated at 37°C for 30 minutes. Post-incubation, the samples underwent centrifugation at 13,400 rpm for 1 minute, followed by discarding of 900 μL of supernatant. Subsequently, 900 μL of phosphate-buffered saline (PBS) was added and thoroughly mixed, after which centrifugation at 13,400 rpm for 1 minute was repeated. This washing procedure was iterated thrice, leaving behind 100 μL of bacterial solution during the final cycle. PBS was judiciously added to adjust the bacterial concentration to 50 × 10^6 cells per milliliter. Finally, 85 μL of the bacterial solution was combined with 15 μL of OptiPrep (Sigma, D1556) and meticulously mixed. For each experiment, a lysis reagent mixture totaling 240 μL was prepared, comprising 30 μL of green buffer (prepGEM Bacteria, PBA 0100), 3 μL of lysozyme (prepGEM Bacteria, PBA 0100), 3 μL of prepgem (prepGEM Bacteria, PBA 0100), 3 μL of lysostaphin (1 mg/mL in 20 mM sodium acetate, pH 4.5, Sigma, L7386), 6 μL of 20 mg/mL bovine serum albumin (B14, Thermo Fisher Scientific), 3 μL of 100 μM random hexamer with the last two 3’ end bases phosphorothioated (ordered from Sangon Biotech), and 192 μL of water. The microbial suspension was introduced into a 2 mL syringe (KDL 2-mL syringe) and connected to the microbial suspension device inlet via a needle (Straight syringe needles, KangHong) and polyethylene tubing (BB31695-PE/2, Scientific Commodities, Inc.). Similarly, the lysis reagents and oil surfactant (RAN Biotechnologies, 008-FluoroSurfactant) in HFE 7500 (3M) were connected to the device. Flow rates of 75 μL/h, 300 μL/h, and 500 μL/h were respectively applied for the microbial suspension, lysis reagents, and oil. Droplets collected from the device outlet were transferred into a PCR tube, and the oil at the bottom was replaced with 100 μL of 5% (w/v) oil. Additionally, 60 μL of mineral oil (MI500, Spectrum Chemical MFG Corp.) was added atop the emulsion to prevent evaporation of the aqueous phase within the droplets. Subsequently, most of the bottom oil was removed, and incubation was carried out to facilitate microbial lysis within the droplets. The lysis program comprised incubation at 37°C for 30 minutes, followed by 75°C for 15 minutes, and finally, 95°C for 5 minutes, with subsequent sample storage at 4°C.

**Whole-genome amplification**

In the process of whole-genome amplification, a 100 μL Multiple Displacement Amplification (MDA) mix was meticulously prepared for each experimental run. This mix comprised 16 μL of 10X phi29 DNA Polymerase Buffer (Lucigen, 30221-1), 0.5-2 μL of 100 μM random hexamers with the last two 3' end bases phosphorothioated (IDT), 2 μL of 25 mM deoxynucleotide triphosphates (dNTPs) (Thermo Scientific, R1121), 8 μL of phi29 DNA Polymerase (Lucigen, 30221-1), 2 μL of 20 mg/mL bovine serum albumin (BSA, B14, Thermo Fisher Scientific), 1.6 μL of 10% Tween-20 (diluted from Tween-20, Sigma-Aldrich, P9416-50mL), and 0.8 μL of T2, with the addition of water to achieve a total volume of 100 μL. The droplet emulsion was then transferred to a syringe and reinjected into an M1 device (Mobi Drop). Simultaneously, within the same device, a separate droplet maker was utilized to generate droplets encapsulating the MDA reagents. The synchronization of sample droplet reinjection and reagent droplet generation facilitated the formation of droplet pairs. Applying electric fields ranging from 50 to 200 V at a frequency of 10 kHz through a pair of electrodes, each droplet pair was merged to introduce MDA reagents. Flow rates of 50 μL/h and 75 μL/h were respectively employed for sample droplets and MDA reagents. Subsequent to droplet manipulation, incubation was performed to initiate the amplification of microbial genomes. The MDA incubation protocol entailed maintaining a temperature of 30°C for 8 hours, followed by a brief incubation at 65°C for 10 minutes, with the resultant samples stored at 4°C for further analysis.

**Tagmentation**

In the process of tagmentation, a 90 μL Nextera mix was meticulously prepared for each experimental iteration. This mixture comprised 24 μL of 5X Tagment Buffer L (Vazyme), 30 μL of Tagment DNA Enzyme B (Vazyme), 30 μL of Tagment DNA Enzyme C (Vazyme), 1.8 μL of 20 mg/mL bovine serum albumin (BSA, B14, Thermo Fisher Scientific), and 4.2 μL of water.Sample droplets were merged with droplets containing commercially available tagmentation reagents (Nextera) using the M1 device. Flow rates of 25 μL/h and 75 μL/h were respectively employed for sample droplets and tagmentation reagents. Following droplet manipulation, incubation ensued to facilitate the tagmentation of DNA products. The tagmentation incubation protocol involved maintaining a temperature of 55°C for 10 minutes, after which the samples were stored at 10°C for subsequent analysis.

**Barcoding**

In the PCR barcoding process, a 420 μL PCR mix was meticulously prepared for each experimental run. This mixture consisted of 84 μL of water, 24 μL of 10 μM Reverse primer (MS-PCR-reverse, Sangon Biotech), 6 μL of 20 mg/mL bovine serum albumin (BSA, B14, Thermo Fisher Scientific), and 6 μL of 10% Tween-20 (diluted from Tween-20, Sigma-Aldrich, P9416-50mL). Sample droplets were merged with droplets containing PCR reagents and a barcoding bead using the M1 device. Flow rates of 50 μL/h, 25-50 μL/h, and 350 μL/h were respectively applied for sample droplets, beads, and PCR reagents. Following droplet manipulation, barcoding of DNA within the droplets was facilitated. Bar code oligos were released from the beads by exposing droplets to UV light (365 nm at ~10 mW/cm2, Black Ray Xenon Lamp) for 10 minutes. Subsequently, PCR was performed to barcode the DNA within the droplets. The PCR barcoding protocol entailed incubation at 72°C for 4 minutes, followed by an initial denaturation step at 98°C for 30 seconds. This was succeeded by 10 cycles of denaturation at 98°C for 7 seconds, annealing at 60°C for 30 seconds, and extension at 72°C for 40 seconds. A final extension step at 72°C for 5 minutes concluded the process, with the resultant samples stored at 4°C for further analysis.

**Sequencing library buliding**

Following PCR, the emulsion of droplets is initially disrupted by introducing 200 μL of 20% (v/v) PFO (1H,1H,2H,2H-Perfluoro-1-octanol, 370533 Sigma Aldrich) in HFE 7500 (3M) into each sample. Subsequently, the aqueous phase is purified using 1.2X volume of AMPure beads (A63881, Beckman Coulter), and the resulting DNA is resuspended in 32 μL of water (Thermo Fisher Scientific, 10977023). For sequencing purposes, PCR is utilized to append sequencing adapters (Illumina) and a sample index (TruSeq index) to each purified DNA sample, thereby enabling the sequencing of multiple samples within a single sequencing run. A pre-amplification reaction reagent of 14.5 μL is prepared for each sample, comprising 12.5 μL of 2x pre-amplification mix, 1 μL of Ms-PCR-P5-Full, and 1 μL of primer. Subsequently, 10.5 μL of DNA sample is combined with the pre-amplification reaction reagent. The pre-amplification protocol involves incubation at 98°C for 30 seconds, followed by 8 cycles of denaturation at 98°C for 15 seconds, annealing at 60°C for 30 seconds, and extension at 72°C for 5 minutes. Following incubation, the samples are stored at 4°C until further analysis.

**Purification of the pre-amplified products**

After the addition of 0.8X AMPure beads to the centrifuge tube containing the samples, they are thoroughly mixed and incubated at room temperature for 5 minutes. Subsequently, the supernatant is discarded after placing the tubes on a magnetic rack for 5 minutes. Following this, 200 μL of freshly prepared 80% ethanol is added to clean the beads, and the process is repeated once. The AMPure beads are then allowed to dry, and 25 μL of ultra-pure water is added, followed by incubation at room temperature for 5 minutes. After incubation, the tubes are placed on a magnetic stand for 5 minutes, and 23 μL of the samples are transferred to a 200 μL centrifuge tube. For purification of the 0.6X pre-amplified product samples, 10 μL of the 0.8X pre-amplification purification product is transferred to a 0.2 mL centrifuge tube, and 20 μL of water is added. The remaining samples are stored at -20°C before sequencing. Subsequently, the appropriate volume of sample is taken based on the concentration of the 0.6X pre-amplified product samples, and water is added to replenish the volume to 26 μL. For fragmentation, a 9 μL fragmenting reagent is prepared for each sample, comprising 7 μL of Fragmentation buffer and 2 μL of fragmentation enzymes (NEB). The sample is mixed with the fragmenting reagent, and the incubation program involves maintaining a temperature of 37°C for 5 minutes, followed by a brief incubation at 65°C for 30 seconds, with the resultant samples stored at 4°C. Similarly, a 33.5 μL linking reagent is prepared for each sample, comprising 30 μL of linking buffer, 1 μL of oligase, and 2.5 μL of the specified working concentration of connector. The sample is mixed with the linking reagent, and the incubation program entails maintaining a temperature of 20°C for 15 minutes, with the samples stored at 4°C thereafter.

**Separation of connection products**

After adding 69 μL of NF-H2O to the amplification product and introducing 0.3X AMPure beads, the mixture is incubated at room temperature for 5 minutes. Following this, the supernatant is transferred to a new centrifuge tube after placing it on a magnetic rack for 5 minutes. Subsequently, 0.3X AMPure beads are added to the centrifuge tube, and the mixture is incubated at room temperature for 5 minutes. The supernatant is then discarded after placing the tube on a magnetic rack for 5 minutes. To clean the AMPure beads, 1 mL of freshly prepared 80% ethanol is added, and the process is repeated once. The beads are allowed to dry, after which 22 μL of ultra-pure water is added, and the mixture is incubated at room temperature for 5 minutes. After 5 minutes on a magnetic stand, 20 μL of the samples are transferred to a 200 μL centrifuge tube. For library tag amplification, a 30 μL library tag amplification reagent is prepared for each sample, comprising 25 μL of Amplification mixes, 2.5 μL of Amplification primer 1 (Illumina S50X), and 2.5 μL of Amplification primer 2 (Illumina N70X). The sample is mixed with the library tag amplification reagents, and the incubation program involves maintaining a temperature of 98°C for 30 seconds, followed by 11 cycles of denaturation at 98°C for 10 seconds and annealing/extension at 65°C for 75 seconds. Subsequently, a final extension step at 65°C for 5 minutes concludes the process, with the samples stored at 4°C until further analysis.

**Sorting of library fragments**

Following amplification, 50 μL of NF-H2O is added to the amplification product, and 0.5X AMPure beads are introduced. The mixture is thoroughly mixed and incubated at room temperature for 5 minutes. After incubation, the supernatant is transferred to a new centrifuge tube after placing it on a magnetic rack for 5 minutes. Subsequently, 0.3X AMPure beads are added to the centrifuge tube containing the supernatant, and the mixture is again thoroughly mixed and incubated at room temperature for 5 minutes. After this, the supernatant is discarded after placing the tube on a magnetic rack for 5 minutes. To clean the AMPure beads, 1 mL of freshly prepared 80% ethanol is added, and the process is repeated once. The beads are allowed to dry, and then 22 μL of ultra-pure water is added to the beads. The mixture is incubated at room temperature for 5 minutes. After 5 minutes on a magnetic stand, 20 μL of the samples are transferred to a 200 μL centrifuge tube.

**Raw data process**

The data underwent preprocessing steps using Fastp (version 0.23.2) and Cutadapt (version 4.1) software tools. These tools were employed to eliminate adaptors, sequences of low quality, and short sequences from the dataset. Following this initial processing, an in-house Python script was utilized to detect the barcodes present in each read and subsequently segregate the reads into separate FASTQ files based on their respective barcodes.

**Co-assemble of species genomes**

The de novo assembly of genomes from the reads of each of the XX Single Amplified Genomes (SAGs) was performed using SPAdes software[1] (version 3.13.0) with the parameters “--sc –careful”. Subsequently, Contamination and Completeness metrics were estimated for each assembly result using CheckM[2]. To compute and compare signatures of these assembled genomes, Sourmash[3] (version 2.0.0) was employed with a k-mer size of 51 and default settings, generating a matrix of estimated similarities between each genome. Assembled genomes exhibiting a similarity score of more than 50 were deemed as over-assembled and subsequently removed from further co-assembly processes. A hierarchical clustering method was employed using SciPy (version 1.1.0), with the following parameters: method=complete, metric=Euclidean, criterion='inconsistent', and threshold=0.95. This method facilitated the grouping of SAGs into bins. Should any new bin contain more than 15 SAGs, it was split into smaller groups of 15 SAGs each. A threshold of 0.95 was verified to ensure conservative bin grouping, thereby minimizing the improper grouping of SAGs from different species. Subsequently, all reads within each bin were utilized to co-assemble a tentative genome, and similarities between these tentative genomes were compared. The bins were then clustered based on these comparisons. This iterative process continued until bins with more than 50% completeness constituted more than 50% of the total bins.

To segregate bins potentially containing Single Amplified Genomes (SAGs) from multiple species, we assessed contig alignment patterns. Reads from each SAG were aligned to the de novo co-assembled genome from the respective bin using bowtie2 (version 2.2.5) with default parameters[4]. For each contig in the tentative genome surpassing 1000 base pairs, a vector was constructed, indicating the number of reads aligned to the contigs from each SAG. A hierarchical clustering method, specifically the ward method with default parameters, was employed to group vectors of contigs into two distinct clusters. For each SAG, if more than 95% of aligned reads were aligned to one of the two groups of contigs, the SAG was designated as associated with that particular group of contigs. This binary splitting process continued iteratively until one of the following conditions was met: more than 60% of the SAGs were excluded from the current bin, both resulting new bins contained fewer than 10 SAGs, or the difference in SAGs between the resulting new bin and the current bin was less than 3. To amalgamate bins representing the same species for genome assembly, we employed fastANI (version 1.33)[5] with default parameters to compute the average nucleotide identity (ANI) between all pairs of bins. We utilized the commonly-used ANI threshold of >95%, which signifies that two genomes are deemed to represent the same species if their ANI exceeds this threshold. Based on the ANI comparisons, bins identified as representing the same species were combined, and their genomic sequences were assembled. In the final step of genome refinement, contigs shorter than 500 base pairs in each assembled genome were removed. Additionally, to further mitigate the presence of contigs potentially originating from other species within each genome, a normal distribution was fitted using the coverage of contigs on a logarithmic scale. Contigs with coverages exceeding two standard deviations away from the mean of the distribution were subsequently removed. Genomes meeting specific criteria were classified based on their completeness and contamination levels. Those with more than 90% completeness and less than 5% contamination were categorized as high quality, while those with more than 50% completeness and less than 10% contamination were deemed medium quality. To identify the closest species for all high and medium-quality bins, GTDB-TK was employed. This tool facilitated the taxonomic classification of these genomes based on their genomic content.

**Phylogeny analysis of genomes**

The phylogeny of high or medium quality bins was constructed using Anvi'o[6]. Amino acid sequences of six ribosomal proteins (Ribosomal_L1, Ribosomal_L2, Ribosomal_L3, Ribosomal_L4, Ribosomal_L5, and Ribosomal_L6) were extracted, concatenated, and utilized in the analysis. The resulting phylogenomic tree was visualized using ggtree[7].

**Differentiating strains of the same species**

Within each species with high- or medium-quality species-level genomes, each Single Amplified Genome (SAG) was aligned to the assembled genome. Bcftools (version 9) was utilized, employing mpileup with filters for SNPs and %QUAL>30, to identify high-quality single-nucleotide polymorphism (SNP) mutations. SAGs with less than 2 reads aligned to a SNP, as well as those where fewer than 99% of their reads matched at a SNP, were designated as unknown or unaligned at that location. SNPs with fewer than 5% of SAGs aligned to the location, and SNPs where fewer than two SAGs exhibited the reference allele or mutation allele, were removed. Additionally, any SNP with less than 1% of SAGs displaying the reference allele or mutation allele was eliminated. Furthermore, any SAG covering less than 1% or fewer than 10 of the retained SNP locations was removed from further analysis.

To identify the number of strains within each species in our samples, we constructed a dendrogram of Single Amplified Genomes (SAGs) using hierarchical clustering with the method set to 'ward' and utilizing SNP vectors derived from all SAGs. While the exact number of clusters may not be readily apparent from the dendrogram, a sequence of SAGs containing similar SNP sequences can be discerned, indicating their proximity. By comparing the similarities of SNP vectors between SAGs at their shared SNP locations and generating a similarity heatmap with SAGs ordered in the same sequence as the corresponding dendrogram, block-diagonal squares in the heatmap can be observed. These squares indicate that SAGs within each square are more closely related to each other than to SAGs in other squares. Subsequently, SAGs can be re-assigned to strain-level bins based on this information. The UMAP[8] (default parameters) was applied to the SNP data to create dimensional-reduction plots.

To eliminate SAGs that harbor reads from microbes of multiple strains, we constructed the consensus genotype of each strain by comparing the SNP vectors of SAGs belonging to the same strain. If more than 90% of the values at a SNP location from all SAGs within the strain are identical, we utilized this value for the SNP in the consensus genotype for the strain; otherwise, we excluded this SNP location for this strain. We compared the SNP vector of each SAG to the consensus genotype of each strain and assigned strains to those SAGs that matched more than 95% of locations in the consensus genotype of only one strain. This process resulted in the exclusion of fewer than approximately 1% of the SAGs from each species.Subsequently, we co-assembled strain-resolved genomes using reads from all SAGs assigned to each strain with SPAdes, employing default parameters.

**Horizontal gene transfer analysis**

To detect Horizontal Gene Transfer (HGT) events, we searched for blocks of DNA sequences shared by a pair of strain-resolved genomes that are longer than 5000 base pairs and exhibit more than 99.98% sequence identity. This analysis was performed using blastp (version 2.5.0). To filter out HGT sequences potentially arising from contaminated Single Amplified Genomes (SAGs), we selected all SAGs from each strain-resolved genome and aligned reads from each SAG to the corresponding strain-resolved genome. SAGs with an overall sequence alignment ratio below 90% were removed to mitigate the influence of potential contamination. The possibility of HGT events occurring due to contamination was calculated following the method described in Zheng et al., Science. To further validate the remaining detected HGT sequences, we aligned reads from all the filtered SAGs from both HGT-associated species. We calculated the number of SAGs belonging to each strain-resolved genome with more than 500 base pairs coverage over the HGT sequence. We then explored the statistical likelihood of the observed fraction of SAGs containing reads covering the HGT sequence. We constructed a null model under the assumption that if an artifactual HGT event between species A and B is detected, the sequence actually only exists in the genome of species B but appears in the SAGs of species A due to contamination. We assumed a worst-case scenario contamination rate of 20% of SAGs for any strain and species. Under these assumptions, the upper limit for the probability that any SAG from species A is contaminated by species B was calculated as: 0.2 * (Nb / N), where Nb is the number of SAGs from species B, and N is the total number of SAGs. If the observed number of SAGs for species A is Na, and the observed number of SAGs contaminated by B is up to x, then the probability that equal to or more than x of the SAGs from species A are contaminated by species B was calculated as: 1-binom.cdf(x, Na, 0.2 * Nb / N). The genes on HGT sequences were predicted using prokka (version 1.14.5)[9].

**Sequencing of metagenomic DNA**

The genomic DNA was fragmented using the Frag enzyme, resulting in 350bp-sized fragments. These fragments underwent end-polishing, A-tailing, and adaptor ligation for DNB sequencing, followed by PCR amplification. The PCR product was heat-denatured with a complementary molecule and then ligated using DNA ligase. The remaining linear molecule was digested with exonuclease, producing a single-strand circular DNA library. Library quality was assessed using Qubit for quantification, real-time PCR, and a bioanalyzer for size distribution analysis. Quantified libraries were evenly pooled to form DNA Nanoballs (DNB), which were subsequently sequenced on a DNBseq-T7 platform with a PE150 read length, yielding 20 G of raw data per sample.

**Assembly of metagenomes, prediction of genes, taxonomic assignments, and statistical analysis**

Trimmomatic (version 0.39) was utilized for initial preprocessing and quality control of the raw sequences. This involved the removal of adapter sequences, reads shorter than 36 bases, and reads with quality scores below 15. To eliminate host contamination from the shotgun metagenomic data, KneadData (v0.7.7) (https://github.com/biobakery/kneaddata) was employed. The cleaned metagenomic reads were then aligned against marker genes from microbial reference genomes using Bowtie2 (version 2.5.1), facilitated by the MetaPhlAn4 package. Assembly of the clean reads was performed using megahit v1.2.9[10], utilizing default parameters[11, 12]. MetaQUAST [13]v5.0.2 was subsequently used to evaluate all metagenome assemblies, providing statistical data such as N50 and the number of assembled contigs. MetaPhlan4 was employed for taxonomic classification and abundance estimation. Clean reads were assembled using megahit v1.2.9 using default parameters. NGS reads were assembled using metaSPAdes v.3.15.5 and default parameters [11, 12]. Binning of metagenomic contigs was conducted using Maxbin 2.0[14], MetaBAT2 [15] and CONCOCT[16] embedded in metaWRAP v1.3[17], with default parameters. A refinement step was then performed using the bin_refinement module from MetaWRAP to combine and improve the results generated by the three binners, the cutoff value of genome completeness was set at 50% and that of contamination was 10%. Self-mapping was conducted by Bowtie2[18] and Samtools[19]. The refined bins generated for contigs from each metagenome assembly methods was subsequently dereplicated with dRep v2.3.2[20] to extract the MAGs that display the best quality and represent individual metagenomic species. The lineage, completeness and contamination level of the recovered metagenome-assembled genomes (MAG) were estimated using CheckM v1.2.2[2], with lineage-specific marker genes as reference. Metagenome-assembled genomes (MAGs) were assigned to species-level genome bins (SGBs) and reconstructed into a phylogenetic tree by PhyloPhlAn 3.0[21]. GTDB-Tk v2.1.0 was used to assign taxonomic classifications of MAGs. All phylogenetic trees were visualized and modified in iTOL[22].
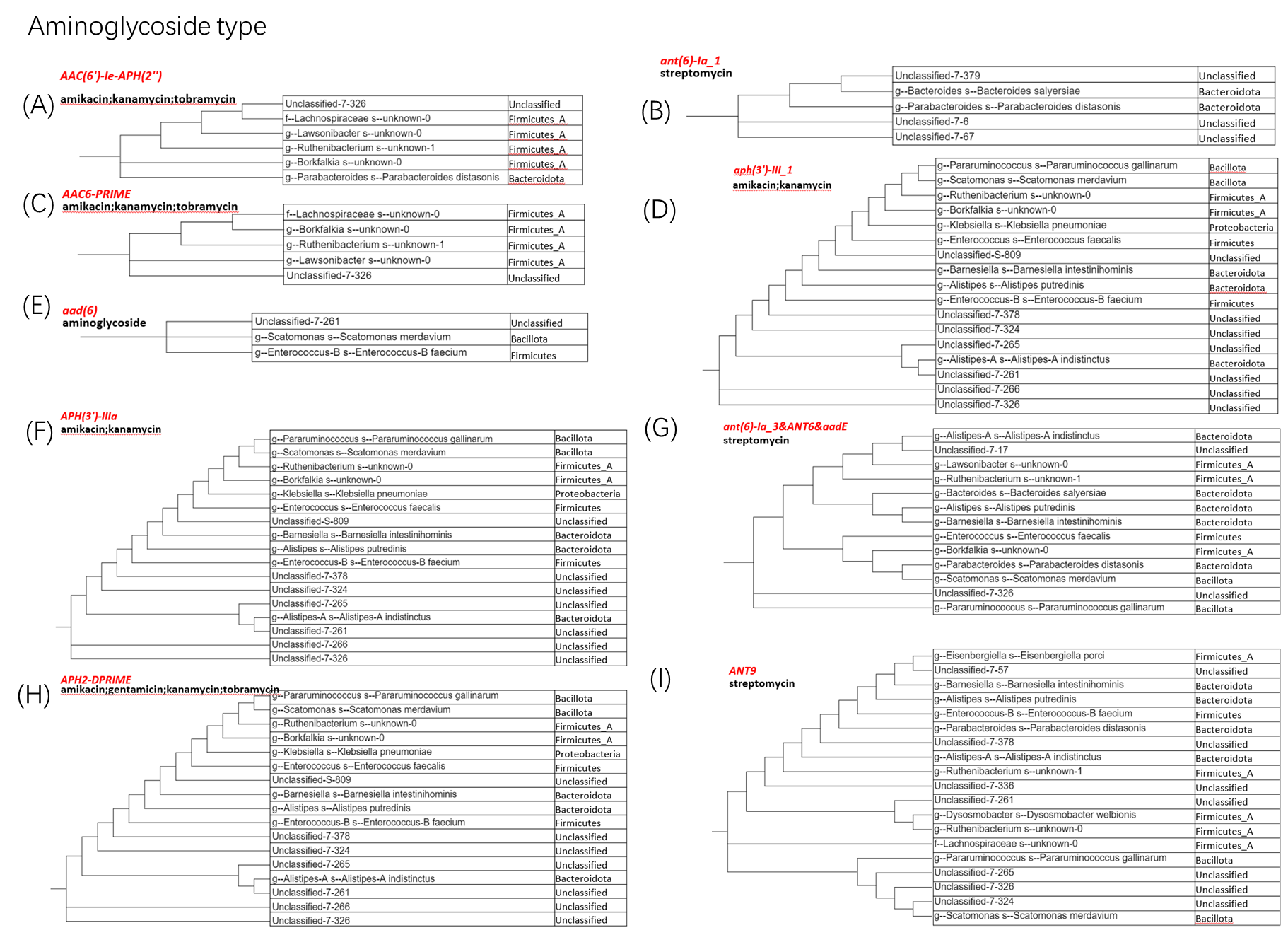


**Figure S1: Co-evolutionary phylogenetic analysis of aminoglycoside resistance genes in bacteria.** It presents phylogenetic trees of various aminoglycoside resistance genes derived from different bacterial species. Each panel, from A to I, represents a distinct aminoglycoside resistance gene, showcasing the diversity and variants of each gene among various bacterial strains. Specifically, (A) represents AAC(6’)-Ie-APH(2’‘), (B) shows ant(6)-Ia_1, (C) depicts AAC6-PRIME, (D) illustrates aph(3’)-III_1, (E) presents aad(6), (F) highlights APH(3’)-IIIa, (G) demonstrates ant(6)-Ia, (H) portrays APH2-DPRIME, and (I) exhibits ANT9. The trees are constructed with lines connecting names of bacterial strains or species, indicating their genetic relationships.

**
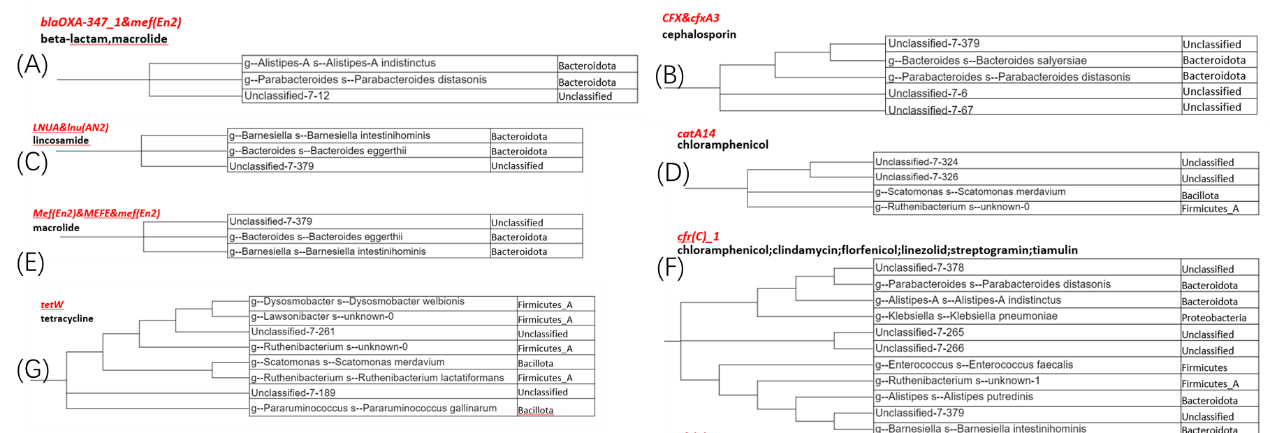
Figure S2: Phylogenetic analysis of antibiotic resistance genes in bacteria.** It presents the phylogenetic trees of various antibiotic resistance genes derived from different bacterial species. From A to G represents a distinct antibiotic resistance gene, showcasing the diversity and variants of each gene among various bacterial strains. (A) represents blaOXA – 347,(B) showcases cfxA3,(C) depicts lnu(AN2),(D) illustrates catA14; (E) presents Mef(En2); (F) highlights cfr(C)_1; (G) demonstrates tet(W). The trees are constructed with lines connecting names of bacterial strains or species, indicating their genetic relationships. This analysis provides a comprehensive view of the co-evolution and distribution of antibiotic resistance genes in bacteria, aiding in understanding the spread and evolution of antibiotic resistance. The figure provides a detailed view of the co-evolution and distribution of antibiotic resistance genes in bacteria.


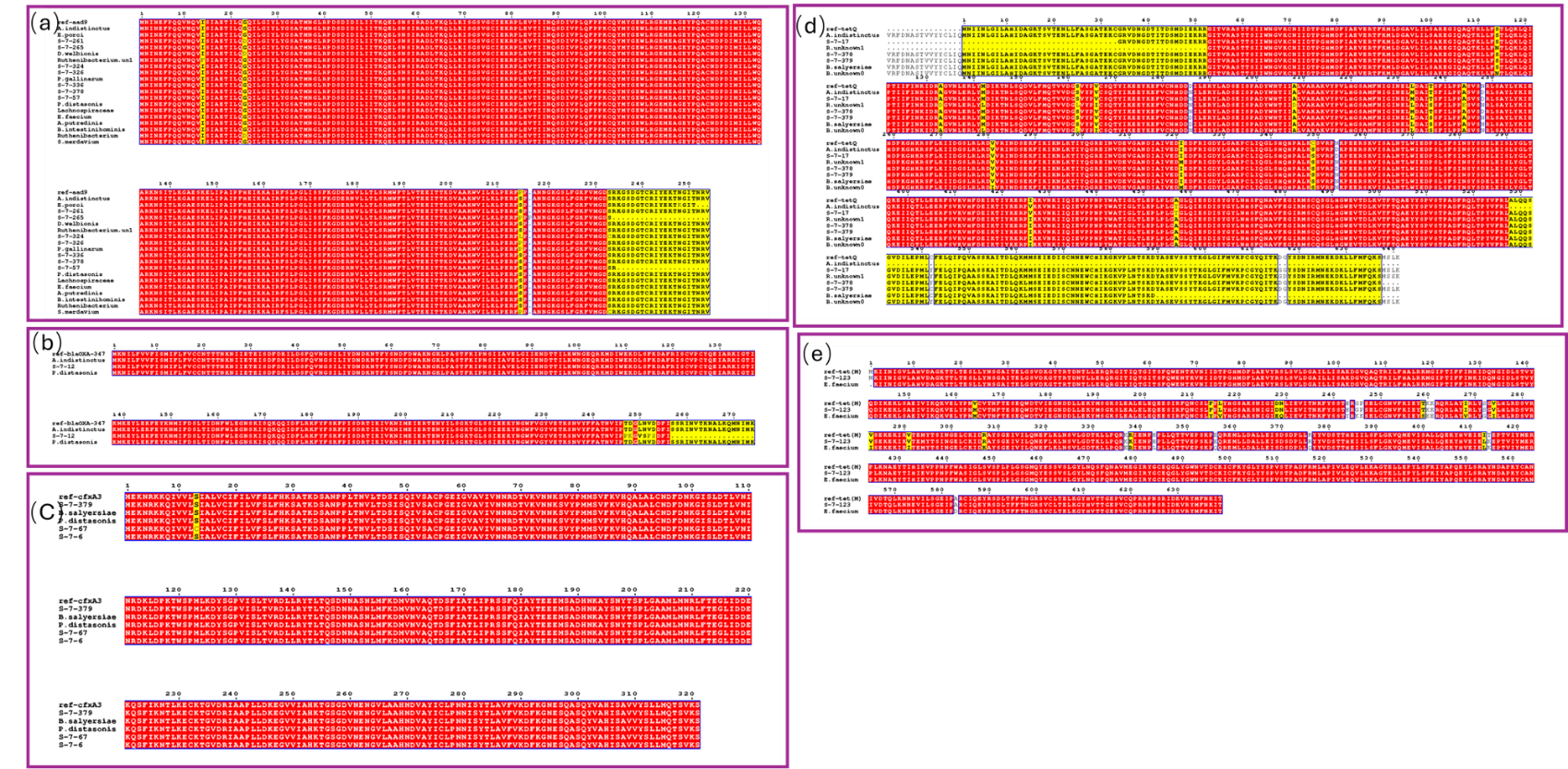


**Figure S3**:**Comparative amino acid alignment of key antibiotic resistance genes reveals conserved functional domains and mutation hotspots.** Alignments display the sequence of a reference resistance gene (top row of each panel) compared to variants identified from single-cell genomic data in this study. Analyzed genes include: **(a)** *aad9* (aminoglycoside 3′-adenylyltransferase, confers resistance to streptomycin and spectinomycin), **(b)** *bla*_OXA-347_ (class D beta-lactamase, confers resistance to carbapenems), **(c)** *cfxA3* (class A beta-lactamase, confers resistance to cephalosporins), **(d)** *tet(Q)* (ribosomal protection protein, confers tetracycline resistance), and **(e)** *tet(M)* (ribosomal protection protein, confers tetracycline resistance). Red residues: Strictly conserved amino acids, indicating structurally or catalytically essential sites (e.g., active site motifs, ATP-binding regions in aad9, or key catalytic residues in beta-lactamases).Yellow residues: Non-conservative or conservative amino acid substitutions, indicating potential functional divergence, neutral drift, or adaptive mutations.

Reference

1. Prjibelski, Andrey, Dmitry Antipov, Dmitry Meleshko, Alla Lapidus, Anton Korobeynikov. 2020. “Using SPAdes de novo assembler.” *Current protocols in bioinformatics* 70: e102. <https://doi.org/10.1002/cpbi.102>

2. Parks, Donovan H, Michael Imelfort, Connor T Skennerton, Philip Hugenholtz, Gene W Tyson. 2015. “CheckM: assessing the quality of microbial genomes recovered from isolates, single cells, and metagenomes.” *Genome research* 25: 1043-1055. <https://doi.org/10.1101/gr.186072.114>

3. Brown, C Titus, Luiz Irber. 2016. “sourmash: a library for MinHash sketching of DNA.” *Journal of open source software* 1: 27. <https://doi.org/10.21105/joss.00027>

4. Langdon, William B. 2015. “Performance of genetic programming optimised Bowtie2 on genome comparison and analytic testing (GCAT) benchmarks.” *BioData mining* 8: 1-7. <https://doi.org/10.1186/s13040-014-0034-0>

5. Jain, Chirag, Luis M Rodriguez-R, Adam M Phillippy, Konstantinos T Konstantinidis, Srinivas Aluru. 2018. “High throughput ANI analysis of 90K prokaryotic genomes reveals clear species boundaries.” *Nature communications* 9: 5114. <https://doi.org/10.1038/s41467-018-07641-9>

6. Eren, A Murat, Özcan C Esen, Christopher Quince, Joseph H Vineis, Hilary G Morrison, Mitchell L Sogin, Tom O Delmont. 2015. “Anvi’o: an advanced analysis and visualization platform for ‘omics data.” *PeerJ* 3: e1319. <https://doi.org/10.7717/peerj.1319>

7. Yu, Guangchuang, David K Smith, Huachen Zhu, Yi Guan, Tommy Tsan‐Yuk Lam. 2017. “ggtree: an R package for visualization and annotation of phylogenetic trees with their covariates and other associated data.” *Methods in Ecology and Evolution* 8: 28-36. <https://doi.org/10.1111/2041-210X.12628>

8. Becht, Etienne, Leland McInnes, John Healy, Charles-Antoine Dutertre, Immanuel WH Kwok, Lai Guan Ng, Florent Ginhoux, Evan W Newell. 2019. “Dimensionality reduction for visualizing single-cell data using UMAP.” *Nature biotechnology* 37: 38-44. <https://doi.org/10.1038/nbt.4314>

9. Seemann, Torsten. 2014. “Prokka: rapid prokaryotic genome annotation.” *Bioinformatics* 30: 2068-2069. <https://doi.org/10.1093/bioinformatics/btu153>

10. Li, Dinghua, Chi-Man Liu, Ruibang Luo, Kunihiko Sadakane, Tak-Wah Lam. 2015. “MEGAHIT: an ultra-fast single-node solution for large and complex metagenomics assembly via succinct de Bruijn graph.” *Bioinformatics* 31: 1674-1676. <https://doi.org/10.1093/bioinformatics/btv033>

11. Koren, Sergey, Brian P Walenz, Konstantin Berlin, Jason R Miller, Nicholas H Bergman, Adam M Phillippy. 2017. “Canu: scalable and accurate long-read assembly via adaptive k-mer weighting and repeat separation.” *Genome research* 27: 722-736. <https://doi.org/10.1101/gr.215087.116>

12. Nurk, Sergey, Dmitry Meleshko, Anton Korobeynikov, Pavel A Pevzner. 2017. “metaSPAdes: a new versatile metagenomic assembler.” *Genome research* 27: 824-834. <https://doi.org/10.1101/gr.213959.116>

13. Mikheenko, Alla, Vladislav Saveliev, Alexey Gurevich. 2016. “MetaQUAST: evaluation of metagenome assemblies.” *Bioinformatics* 32: 1088-1090. <https://doi.org/10.1093/bioinformatics/btv697>

14. Wu, Yu-Wei, Blake A Simmons, Steven W Singer. 2016. “MaxBin 2.0: an automated binning algorithm to recover genomes from multiple metagenomic datasets.” *Bioinformatics* 32: 605-607. <https://doi.org/10.1093/bioinformatics/btv638>

15. Kang, Dongwan D, Jeff Froula, Rob Egan, Zhong Wang. 2015. “MetaBAT, an efficient tool for accurately reconstructing single genomes from complex microbial communities.” *PeerJ* 3: e1165. <https://doi.org/10.7717/peerj.1165>

16. Alneberg, Johannes, Brynjar Smári Bjarnason, Ino De Bruijn, Melanie Schirmer, Joshua Quick, Umer Z Ijaz, Leo Lahti, Nicholas J Loman, Anders F Andersson, Christopher Quince. 2014. “Binning metagenomic contigs by coverage and composition.” *Nature methods* 11: 1144-1146. <https://doi.org/10.1038/nmeth.3103>

17. Uritskiy, Gherman V, Jocelyne DiRuggiero, James Taylor. 2018. “MetaWRAP—a flexible pipeline for genome-resolved metagenomic data analysis.” *Microbiome* 6: 1-13. <https://doi.org/10.1186/s40168-018-0541-1>

18. Langmead, Ben, Steven L Salzberg. 2012. “Fast gapped-read alignment with Bowtie 2.” *Nature methods* 9: 357-359. <https://doi.org/10.1038/nmeth.1923>

19. Li, Heng, Bob Handsaker, Alec Wysoker, Tim Fennell, Jue Ruan, Nils Homer, Gabor Marth, Goncalo Abecasis, Richard Durbin, Genome Project Data Processing Subgroup. 2009. “The sequence alignment/map format and SAMtools.” *Bioinformatics* 25: 2078-2079. <https://doi.org/10.1093/bioinformatics/btp352>

20. Olm, Matthew R, Christopher T Brown, Brandon Brooks, Jillian F Banfield. 2017. “dRep: a tool for fast and accurate genomic comparisons that enables improved genome recovery from metagenomes through de-replication.” *The ISME journal* 11: 2864-2868. <https://doi.org/10.1038/ismej.2017.126>

21. Asnicar, Francesco, Andrew Maltez Thomas, Francesco Beghini, Claudia Mengoni, Serena Manara, Paolo Manghi, Qiyun Zhu, Mattia Bolzan, Fabio Cumbo, Uyen May. 2020. “Precise phylogenetic analysis of microbial isolates and genomes from metagenomes using PhyloPhlAn 3.0.” *Nature communications* 11: 2500. <https://doi.org/10.1038/s41467-020-16366-7>

22. Letunic, Ivica, Peer Bork. 2016. “Interactive tree of life (iTOL) v3: an online tool for the display and annotation of phylogenetic and other trees.” *Nucleic acids research* 44: W242-W245. <https://doi.org/10.1093/nar/gkw290>
